# Electronic Modulation of K⁺‐Intercalated Polymeric Carbon Nitride via ─C≡N/─OH Functionalization for Efficient Photocatalytic H_2_O_2_ Production

**DOI:** 10.1002/advs.202521125

**Published:** 2025-12-07

**Authors:** Qingquan Xue, Shuang Liu, Ye Yang, Jiaxiang Li, Fan Tang, Shengdao Shan, Xiaofeng Shen, Hua Pan, Chi He

**Affiliations:** ^1^ Keqiao Science and Technology Innovation Center Zhejiang Shuren University Zhejiang Collaborative Innovation Center for Full‐Process Monitoring and Green Governance of Emerging Contaminants Key Laboratory of Pollution Exposure and Health Intervention of Zhejiang Province Interdisciplinary Research Academy (IRA) Zhejiang Shuren University Hangzhou 310015 P. R. China; ^2^ Key Laboratory of Recycling and Eco‐treatment of Waste Biomass of Zhejiang Province School of Environmental and Natural Resources Zhejiang University of Science and Technology Hangzhou 310023 P. R. China; ^3^ State Key Laboratory of Multiphase Flow in Power Engineering Xi'an Jiaotong University Xi'an Shaanxi 710049 P. R. China

**Keywords:** bridge‐type PCN, ─C≡N/─OH groups, interfacial electron transfer, K^+^ doping, photocatalytic H_2_O_2_ production

## Abstract

Polymeric carbon nitride (PCN) holds great promise for photocatalytic H_2_O_2_ production owing to its excellent stability and structural tunability. However, the inferior overall photocatalytic performance of PCN owing to its limited charge separation efficiency is a longstanding challenge. Herein, a K⁺‐doped bridge‐type PCN featuring ─C≡N and ─OH surface groups is synthesized via thermal polymerization. K‐PCN exhibits superior efficiency for photocatalytic H_2_O_2_ production (8647.78 µM·h^−1^) with a quantum efficiency of 17.92% at 420 nm, remarkably surpassing that of PCN (86.96 µM·h^−1^ and 0.129%). K⁺ ions tend to reside within the interlayers of PCN, where they create K^+^‐bridges that reduce interlayer spacing, strengthen π–π stacking, and improve crystallinity, thereby accelerating charge transport by shortening carrier migration pathways and suppressing recombination. Moreover, K^+^ perturbs the local electronic structure via interaction with nitrogen lone pairs, thereby narrowing the bandgap and enhancing visible‐light absorption. The introduction of ─C≡N and ─OH groups enrich the surface with polar sites, with ─C≡N promoting O_2_ adsorption and activation through electron delocalization and ─OH increasing hydrophilicity and O_2_ concentration via hydrogen bonding. This study elucidates the pathway and mechanism of photocatalytic oxygen reduction and provides novel insights into the reasonable design of bridge‐type group‐modified PCN for efficient H_2_O_2_ production.

## Graphical abstract

K^+^ is successfully embedded in the PCN interlayers via a high‐temperature polymerization. The incorporation of K^+^ perturbs the local electronic structure via interaction with nitrogen lone pairs, narrowing the PCN bandgap, which exhibits superior photocatalytic performance, achieving a high H_2_O_2_ generation rate both in situ and under sunlight, outperforming most modified PCN catalysts.

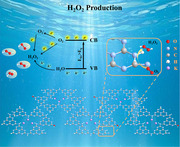

## 1 Introduction

Hydrogen peroxide (H_2_O_2_)—an environmentally friendly oxidant—is widely used in chemical synthesis, environmental remediation, medical disinfection, and fuel cells.^[^ ^1^, ^2^, ^3^, ^4^, ^5^ ^]^ Currently, over 95% of global H_2_O_2_ production relies on the anthraquinone process, which poses certain environmental risks owing to disadvantages such as high energy consumption, process complexity, catalyst loss, and secondary pollution.^[^ ^6^, ^7^ ^]^ Solar‐driven photocatalysis has thus far offered a sustainable and eco‐friendly approach to H_2_O_2_ production by converting oxygen and water into H_2_O_2_ through the oxygen reduction reaction (ORR) or water oxidation reaction (WOR).^[^ ^8^, ^9^, ^10^, ^11^, ^12^, ^13^ ^]^ However, efficient H_2_O_2_ generation remains challenging owing to the rapid recombination of photoinduced charges and difficulty in exciton dissociation in typical photocatalysts.^[^ ^14^, ^15^, ^16^ ^]^ Thus, achieving efficient H_2_O_2_ production requires in‐depth materials research focused on the development of advanced photocatalysts.

Among the numerous available photocatalysts, polymeric carbon nitride (PCN) has attracted significant attention in photocatalysis owing to its unique electronic structure and favorable physicochemical properties.^[^ ^17^, ^18^, ^19^, ^20^, ^21^ ^]^ However, the strong intrinsic Coulombic interactions in PCN generate Frenkel excitons that hinder effective charge separation and exciton dissociation, thus limiting its photocatalytic performance.^[^ ^22^, ^23^ ^]^ Engineering photocatalysts by tailoring their morphology, enhancing their photogenerated charge transfer, and optimizing their active site distribution has largely been achieved through surface defect engineering and elemental doping.^[^ ^24^, ^25^, ^26^, ^27^, ^28^ ^]^ Feng et al.^[^ ^29^ ^]^ synthesized pore‐defective g‐C_3_N_4_ with adequate nitrogen vacancies via the light‐assisted heating method, which exposed more active sites and produced an improvement in photocatalytic capacity. Furthermore, the presence of nitrogen vacancies narrowed the intrinsic bandgap, broadened the light absorption range, and suppressed photogenerated carrier recombination. Zhang et al.^[^ ^30^ ^]^ developed Nv─C≡N─CN with cyano and cyanine defects. The introduction of these functional groups enhanced the electronic structure and improved oxygen adsorption, further contributing to an improved photocatalytic performance. Cao et al.^[^ ^31^ ^]^ successfully synthesized a phosphorus‐doped PCN that enabled dual‐channel water splitting. Notably, the H_2_O_2_ production rate reached 1968 µM·h^−1^ without sacrificing agents. By introducing K^+^ and sulfonylcyanide groups onto carbon nitride, Xiong et al.^[^ ^32^ ^]^ constructed a quasi‐homogeneous photocatalytic system that exhibited an impressive H_2_O_2_ production rate (7886 µmol·g^−1^·h^−1^). The aforementioned investigations demonstrate that rational surface engineering of catalysts serves as an effective strategy to enrich surface‐active sites, thereby facilitating the photocatalytic oxygen reduction to H_2_O_2_. However, in photocatalytic H_2_O_2_ synthesis, both the stepwise single‐electron and the direct two‐electron oxygen reduction pathways compete with the four‐electron reduction to water, hindering the design of catalysts with high two‐electron oxygen reduction reaction (2e^−^ ORR) selectivity.

In this study, a “bridge‐type” carbon nitride photocatalyst (K‐PCN) featuring structural defects and interlayer intercalation was synthesized via the potassium chloride‐assisted calcination of melamine, and its photocatalytic mechanism was systematically investigated. The incorporation of cyano defects (─C≡N) enhances oxygen adsorption by increasing active site density, and intercalated K⁺ ions created interlayer “K^+^‐bridges” that facilitate exciton dissociation. This synergy effect between defect engineering and structural modulation endows K‐PCN with exceptional photocatalytic H_2_O_2_ production activity, achieving a remarkable rate of 8647.78 µM·h^−1^ under continuous O_2_ flow and visible‐light irradiation. Moreover, the system demonstrates the great apparent quantum efficiency (AQY) of 17.92% at 420 nm, highlighting its outstanding light utilization capability. Notably, K‐PCN exhibits exceptional photocatalytic stability, retaining 83.58% of its initial H_2_O_2_ production activity (7231.44 µM·h^−1^) after five consecutive reaction cycles. Under simulated solar irradiation with ambient air flow, the system maintains robust performance, achieving a production rate of 667.11 µM·h^−1^, thus highlighting its potential for practical solar‐driven applications. The positive correlation between H_2_O_2_ generation and the •O_2_^−^ intermediate concentration strongly suggests a sequential single‐electron transport pathway rather than two‐electron reduction. This mechanistic insight not only provides a fundamental understanding of the photocatalytic 2e^−^ ORR process but also establishes new design principles for developing advanced PCNs for efficient H_2_O_2_ production.

## 2 Results and Discussion

### 2.1 Chemical and Band Structures of K‐PCN

**Figure 1:**
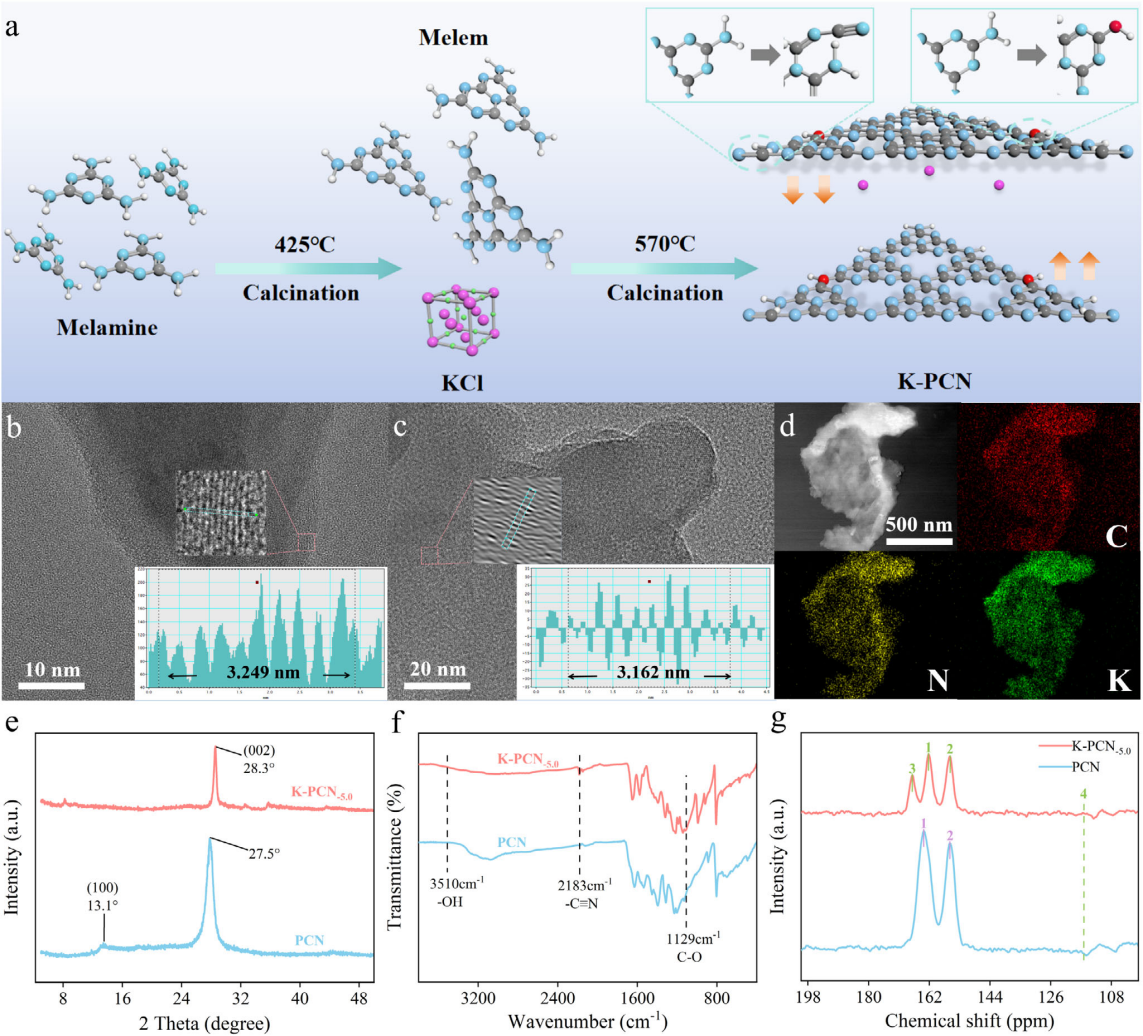
a) Schematic of K‐PCN synthesis; High‐resolution TEM images of b) PCN and c) K‐PCN_‐5.0_; d) Elemental mapping of K‐PCN_‐5.0_; e) XRD pattern and f) FTIR spectra of PCN and K‐PCN_‐5.0_; g) Comparison of solid‐state ^13^C NMR spectra between PCN and K‐PCN_‐5.0_.

PCN was prepared by the conventional thermal polymerization of melamine.^[^ ^33^ ^]^ As illustrated in **Figure** **1**a and K‐PCN_‐x_ (x = 2.5, 5.0, and 7.5, representing the amount of potassium salt) was prepared by grinding Melem with KCl, followed by a high‐temperature polymerization. The incorporation of K^+^ induced a rearrangement charge division between adjacent planes, and the resulting K^+^‐bridged structure exhibited enhanced electronic coupling, facilitating charge transfer and separation. Additionally, the intercalation of K^+^ introduced Lewis acidic sites and reinforced existing Lewis basic sites, thereby promoting hydrogen extraction and proton release.^[^ ^34^ ^]^ The planar structure of the pristine PCN, along with both the planar and spatial configurations of K‐PCN, is shown in Figure S1 (Supporting Information).

The morphologies of the samples were examined using SEM and TEM. PCN had an irregular block structure (Figure S2a, Supporting Information), whereas K‐PCN exhibited a loose and flaky structure (Figure S2b–d, Supporting Information). As depicted in Figure S2e (Supporting Information), compared with pristine PCN, the structure of K‐PCN was observed as a representative ultrathin nanosheet (Figure S2f, Supporting Information). The high‐resolution TEM images shown in Figure 1b,c reveal interplanar lattice spacings of 0.325 and 0.316 nm, corresponding to the (002) planes of PCN and K‐PCN_‐5.0_, respectively. The slightly reduced interlayer spacing in K‐PCN compared to pristine PCN suggests that K⁺ ions act as interlayer bridges, tightening the structure and facilitating more efficient charge transfer between layers.^[^ ^1^ ^]^ Elemental mapping (Figure 1d; Figure S3, Supporting Information) confirms the uniform distribution of C, N, and K, indicating that K⁺ ions are effectively intercalated between the molecular layers. K‐PCN remained well dispersed in water without noticeable sedimentation after 7 days (Figure S4, Supporting Information), demonstrating excellent colloidal stability, which favors the effective adsorption of dissolved oxygen. Moreover, hydrodynamic diameter and zeta potential were explored via dynamic light scattering and Zeta potential analysis. K‐PCN_‐5.0_ had a hydrodynamic diameter of ≈164 nm (Figure S5a, Supporting Information) and a zeta potential of −35.9 mV (Figure S5b, Supporting Information), indicating strong electrostatic repulsion that effectively prevents aggregation. In contrast, the unmodified PCN particles had a larger hydrodynamic diameter (≈190 nm) and a less negative zeta potential (−30.4 mV), which is consistent with their marked propensity for aggregation and sedimentation.

The crystal structures of the prepared samples were analyzed by X‐ray powder diffraction (XRD). As described in Figure 1e and Figure S6a (Supporting Information), the XRD pattern of pristine PCN has two diffraction peaks at 13.1° and 27.5°, corresponding to the (100) and (002) planes, respectively.^[^ ^35^ ^]^ The weak peak at 13.1° is attributed to the in‐plane repeating units of tri‐s‐triazine, whereas the stronger peak at 27.5° corresponds to the interlayer stacking of the conjugated aromatic structure.^[^ ^32^ ^]^ In comparison, the diffraction peaks of K‐PCN slightly shift toward higher angles, indicating a reduction in interlayer spacing owing to K⁺ incorporation. Additionally, the intensity of the (100) peak diminished significantly. The near disappearance of the (100) peak and the weakened (002) reflection suggest that K⁺ doping disrupts the long‐range order of the PCN framework, possibly introducing structural distortion or partial amorphization.^[^ ^36^ ^]^

The fundamental structural changes induced by K⁺ doping were further investigated by FTIR spectroscopy, using pristine PCN as the reference (Figure 1f; Figure S6b, Supporting Information). The extensive absorption band between 2950 and 3500 cm^−1^ can be assigned to the N─H stretching vibrations. Several peaks observed from 1100 to 1700 cm^−1^ result from the characteristic stretching modes of ─C═N and ─C─N in the heterocyclic framework. These results indicate that the incorporation of K⁺ does not significantly adjust the primary melon‐based structure of PCN.^[^ ^31^ ^]^ Notably, a new peak appeared around 2183 cm^−1^, ascribed to the ─C≡N vibrations, indicates the formation of cyano groups during the thermal post‐treatment of PCN with KCl (an observation that distinguishes this work from previous studies).^[^ ^37^ ^]^ Interestingly, a noticeable attenuation of the wide band between 2700 and 3500 cm^−1^ was observed in K‐PCN_‐5.0_, suggesting that the formation of ─C≡N groups likely results from the protonation and subsequent transformation of terminal ─C─NH_2_ groups. In addition, the peaks around 3510 and 1129 cm^−1^ correspond with ─OH and C─O, respectively.^[^ ^37^ ^]^ This observation is further supported by the solid‐state ^13^C NMR spectra. As depicted in Figure 1g, both PCN and K‐PCN_‐5.0_ possess two characteristic peaks at 163.7 and 156.1 ppm, which respectively derive from the NH_X_─*C*─N_2_ (*C1*) and *C*─N_3_ (*C2*).^[^ ^38^ ^]^ However, the C1 peak of K‐PCN shows a noticeable shift compared to that of PCN, indicating a structural modification. This shift further supports the notion that the establishment of ─C≡N is associated with the transformation of terminal ─C─NH_2_ moieties. In addition, two new resonance peaks can be observed in K‐PCN_‐5.0_ at 167.1 and 116.4 ppm, respectively, assigned to the *C3* site (─C≡N) and *C4* site (C─OH), further confirming the formation of cyano and hydroxyl functionalities upon K^+^ modification.^[^ ^39^ ^]^

Compared to PCN, the XPS spectrum of K‐PCN_‐5.0_ exhibited clear signals corresponding to elemental potassium, confirming the successful introduction of K⁺ (Figure S7a, Supporting Information). The valence band (VB) positions for PCN and K‐PCN_‐5.0_ were determined to be 1.78 and 1.93 eV, respectively, according to the XPS‐VB spectra (Figure S7b, Supporting Information). In the C 1*s* spectrum of PCN (Figure S7c, Supporting Information), two distinct peaks were observed at 287.6 and 284.4 eV, deriving from N─C═N and C─C/C═C bonds, respectively.^[^ ^40^ ^]^ In contrast, the C 1*s* of K‐PCN_‐5.0_ revealed two additional peaks at 287.4 and 286.5 eV, attributed to C─O and ─C≡N, respectively. The intensity of the N─(C)_3_ peak at 399.4 eV in the N 1*s* spectrum was notably reduced in K‐PCN, further supporting the successful creation of ─C≡N via modification of the nitrogen coordination environment modification (Figure S7d, Supporting Information). Additionally, in both the C 1*s* and N 1*s* spectra, the N═C─N and ─NH_x_ peaks originally at 287.6 and 400.6 eV shifted to higher binding energies in K‐PCN_‐5.0_. This further confirmed that the formation of ─C≡N groups is closely associated with the protonation and subsequent transformation of terminal ─C─NH_2_ groups. Interestingly, the shift of the ─NH_x_ peak was also related to the formation of the ─OH group through the substitution of O for N.^[^ ^41^ ^]^ As shown in Figure S7e (Supporting Information), the O 1*s* spectrum of PCN exhibited two peaks at 532.3 and 533.9 eV, corresponding to surface hydroxyl groups and adsorbed H_2_O, respectively. In contrast, K‐PCN_‐5.0_ displayed three distinct oxygen species: A peak at 530.8 eV attributed to the O atom in N─C─O, alongside peaks at 532.3 (surface hydroxyl groups) and 533.6 eV (adsorbed H_2_O). Notably, the intensity of the 532.3 eV peak of K‐PCN_‐5.0_ was markedly stronger than that of PCN, indicating a higher density of surface hydroxyl groups, thereby promoting oxygen adsorption and enhancing the catalytic process. The K 2*p* spectrum further confirmed the successful incorporation of K⁺, with a peak at 295.6 eV originating from the K⁺ ions, which acted as interlayer “bridges” within the PCN structure (Figure S7f, Supporting Information). Collectively, the above findings supply convincing proof of the successful synthesis of K⁺‐incorporated carbon nitride.

**Figure 2:**
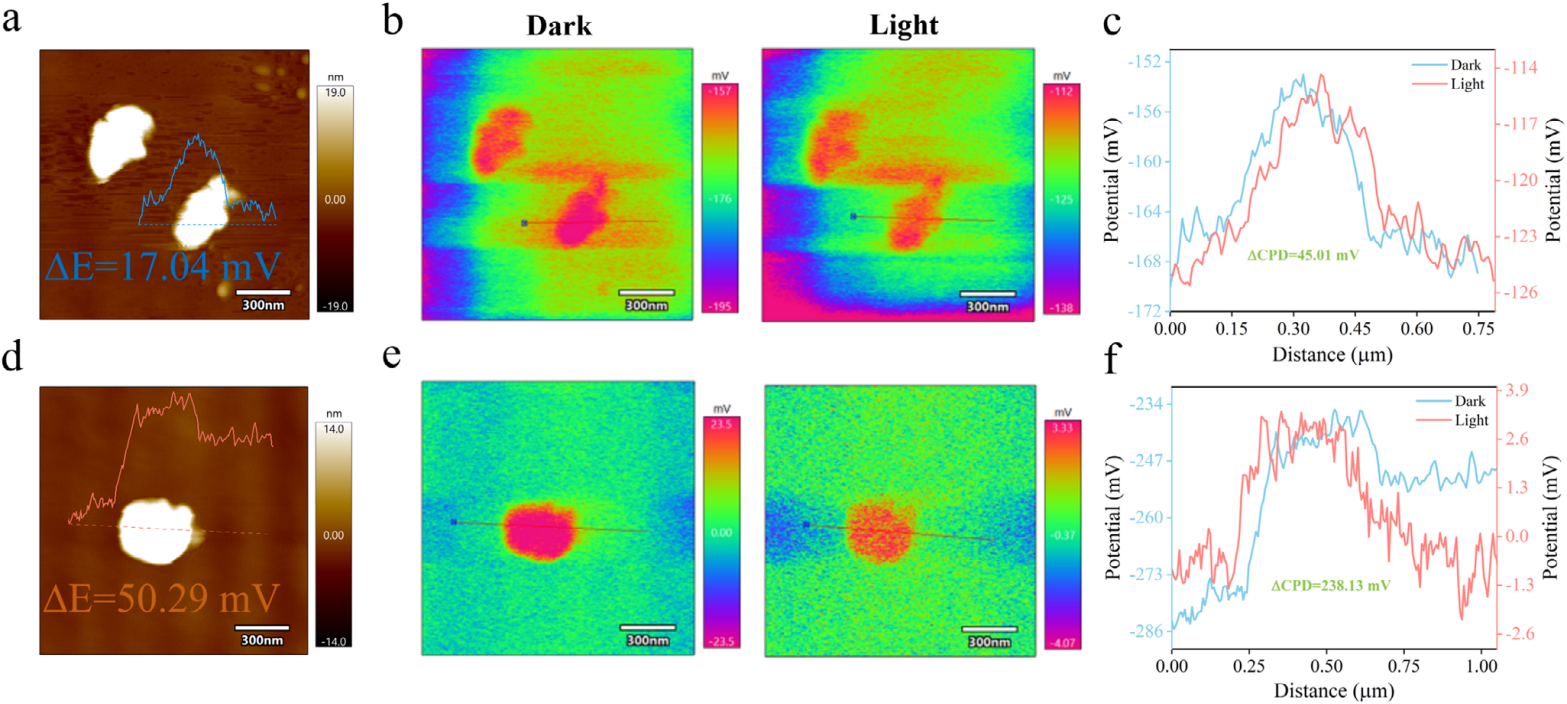
The KPFM images, surface potentials under dark and light conditions, and corresponding CPD profiles of a–c) PCN and d–f) K‐PCN_‐5.0_.

Furthermore, Kelvin probe force microscopy (KPFM) was adopted to evaluate the surface potential allocation of the photocatalysts and to monitor their evolution under light irradiation (**Figure** **2**). K‐PCN_‐5.0_ shows a 2.95‐fold increase in surface photovoltage (ΔE = 50.29 mV; Figure 2d) compared to pristine PCN (ΔE = 17.04 mV; Figure 2a), thereby quantitatively confirming its superior charge‐separation efficiency. This significant improvement was directly characterized by the introduction of cyano group defects and K^+^ interlayer bridges, highlighting their pivotal role in tuning the photogenerated carrier dynamics of PCN. Besides, all samples exhibited significantly increased surface potentials under light irradiation compared to those measured in the dark. This is ascribed to the rapid division of photoinduced electron‐hole pairs in n‐type semiconductors. The contact potential difference (ΔCPD) between the AFM tip and the photocatalyst surface reflects the extent of surface potential enhancement. K‐PCN_‐5.0_ exhibits a ΔCPD of 238.13 mV (Figure 2f), markedly higher than that of PCN (45.01 mV; Figure 2c), directly highlighting its superior photogenerated charge separation capability. The higher ΔCPD value observed for K‐PCN_‐5.0_ indicates the successful establishment of a strong built‐in electric field that significantly enhances the division efficiency of photoinduced carriers and is believed to contribute to the improved photocatalytic H_2_O_2_ production performance.

**Figure 3:**
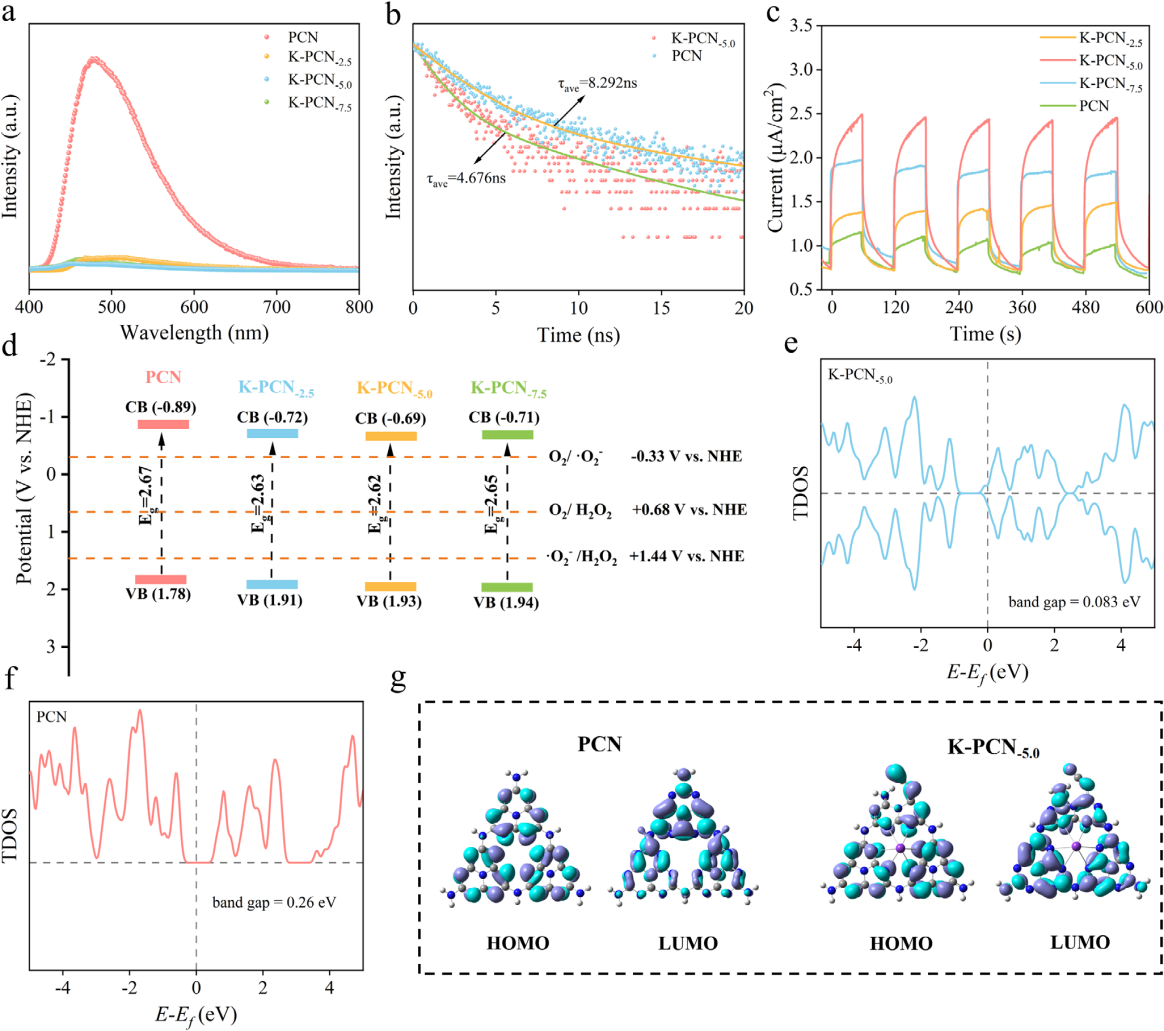
a) PL spectra and b) TRPL spectra of PCN and K‐PCN; c) Transient photocurrent responses of PCN and K‐PCN; d) Band structure of PCN and K‐PCN; DOS of e) K‐PCN_‐5.0_ and f) PCN; g) HOMO and LUMO distributions on PCN and K‐PCN_‐5.0_.

Photoluminescence (PL) spectroscopy was employed to investigate the recombination behavior of the photogenerated electron‐hole pairs in the photocatalysts. As shown in **Figure** **3**a and K‐PCN‐_5.0_ exhibits a markedly lower PL intensity than pristine PCN, indicating significant charge carrier recombination suppression and enhanced exciton dissociation. This effect is likely attributed to K⁺ doping, which facilitates more efficient electron transfer within the material. To further assess the charge transfer dynamics, time‐resolved photoluminescence (TRPL) measurements were conducted to evaluate the kinetics of the photoinduced carriers (Figure 3b; Figure S8 and Table S1, Supporting Information). The fluorescence decay curves were fitted using a double‐exponential model, *R(t) = A_1_ exp(‐t/τ*_1_ *) + A*_2_ *exp(‐t/τ*_2_ *))*, where *A*_1_ and *A*_2_ are the pre‐exponential factors; *τ*_1_ and *τ*_2_ represent the long and short fluorescent lifetimes, respectively. The average lifetime (*τ*_ave_) of the photoinduced charges was calculated using the equation *τ*_ave_ = *(A*_1_ *× τ*_1_ *^2^+A*_2_ *× τ*_2_ *^2^)/(A*_1_ *× τ*_1_ *+ A*_2_ *× τ*_2_). The results indicated that the average fluorescence lifetimes of PCN and K‐PCN_‐5.0_ were 8.292 and 4.676 ns, respectively. The shorter lifetime observed for K‐PCN is attributed to the “bridge” structure formed by K⁺ doping, which facilitates more efficient charge separation and accelerates charge carrier diffusion.^[^ ^34^, ^42^ ^]^ The division and transmission efficiency of photoinduced charge carriers within the 2D conjugated framework of K‐PCN are also key elements influencing its performance. Electrochemical impedance spectroscopy (EIS) results revealed that K‐PCN_‐5.0_ has a smaller arc radius than pristine PCN, indicating lower charge transfer resistance (Figure S9, Supporting Information). This suggests that K^+^ doping effectively enhanced the charge separation and transport efficiency of the modified material. As shown in Figure 3c and K‐PCN_‐5.0_ exhibits a photocurrent response of 2.6 times greater than that of PCN under visible‐light irradiation. This enhancement indicates that the interlayer “bridging” effect of K⁺ facilitated more efficient photoelectron transfer, promoting its rapid involvement in surface redox reactions.^[^ ^43^ ^]^

The light absorption properties of the samples were evaluated by UV–vis DRS. As depicted in Figure S10a (Supporting Information), compared to pristine PCN, K‐PCN_‐5.0_ exhibited enhanced light‐harvesting capability, indicating enhanced visible‐light absorption. Notably, K‐PCN showed a pronounced redshift at its absorption edge, indicating that K⁺ incorporation reduces the bandgap and enhances exciton dissociation efficiency, thereby improving the photocatalyst's light utilization.^[^ ^44^ ^]^ The band gap energies (*E*_g_) were estimated through the Tauc plots rooted in the Kubelka–Munk function (Figure S10b, Supporting Information). The *E*_g_ values were 2.67, 2.63, 2.62, and 2.65 eV for PCN, K‐PCN_‐2.5_, K‐PCN_‐5.0_, and K‐PCN_‐7.5_, respectively, confirming that K⁺ doping slightly narrowed the band gap, consistent with the observed redshift in the absorption edge. The reduction in the band gap of K‐PCN is attributed to the lowering band gap as a result of metal cations doping. The relationship between the band gap energy (*E*_g_), valence band potential (*E*_VB_), and conduction band potential (*E*_CB_) follows the equation: *E*_g_ = *E*_VB_ − *E*_CB_. The *E*_CB_ values can be experimentally determined from the Mott‐Schottky (M‐S) measurements. The positive slopes of the Mott‐Schottky plots (Figure S11, Supporting Information) confirmed that both PCN and K‐PCN behave as n‐type semiconductors. Accordingly, the conduction band potential (*E*_CB_) was estimated to be ≈0.2 eV below the flat band potential (*E*_FB_) according to the intercept of the Mott‐Schottky plots.^[^ ^32^ ^]^ The *E*_CB_ of PCN and K‐PCN_‐x_ were −0.89, −0.72, −0.69, and −0.71 eV, respectively (Table S2, Supporting Information), all of which lie below the O_2_ reduction potential (O_2_/•O_2_^−^, −0.33 V) and the two‐electron O_2_ reduction potential (O_2_/H_2_O_2_, 0.68 V; Figure 3d).

The density of states (DOS) of K‐PCN and PCN were calculated, as shown in Figure 3e,f. The total DOS revealed bandgap values of 0.083 eV for K‐PCN and 0.26 eV for PCN. Although these values are smaller than the experimental results owing to the known tendency of DFT to underestimate band gaps, the trend clearly demonstrates that K⁺ incorporation narrows the band gap. The electronic structures of PCN and K‐PCN_‐5.0_ were explored through DFT calculations to elucidate the relationship between the ─C≡N/─OH functional groups and charge division efficiency.^[^ ^45^ ^]^ The electrostatic potential (ESP) maps of PCN and K‐PCN_‐5.0_ use red and blue to indicate the electron‐rich and electron‐deficient regions, respectively (Figure S12, Supporting Information). PCN exhibited a symmetric electron distribution, thus providing no directional driving force for photogenerated carrier migration and facilitating charge recombination. In contrast, K^+^ doping disrupted this symmetry, separating the positive and negative charge centers and creating a larger molecular dipole. This enhanced polarity promoted more efficient separation of the photogenerated charges. The charge density distributions of the highest occupied molecular orbitals (HOMO) and lowest unoccupied molecular orbitals (LUMO) were also calculated for both the PCN and K‐PCN_‐5.0_ (Figure 3g). Upon light excitation, the photogenerated electrons were promoted from the HOMO to the LUMO orbitals of the catalyst. In PCN, the HOMO and LUMO exhibit symmetrical and uniformly distributed charge densities that facilitate the rapid recombination of photoinduced carriers.^[^ ^46^ ^]^ In contrast, the ─C≡N and ─OH groups in K‐PCN_‐5.0_ disrupt the symmetry, creating a localized charge distribution that leads to distinct spatial separation between the HOMO and LUMO orbitals. This modulation of the electronic band structure facilitates charge excitation and transfer, improves charge separation efficiency, and ultimately boosts the photocatalytic H_2_O_2_ generation.

### 2.2 Photocatalytic Performance of H_2_O_2_ Production

**Figure 4:**
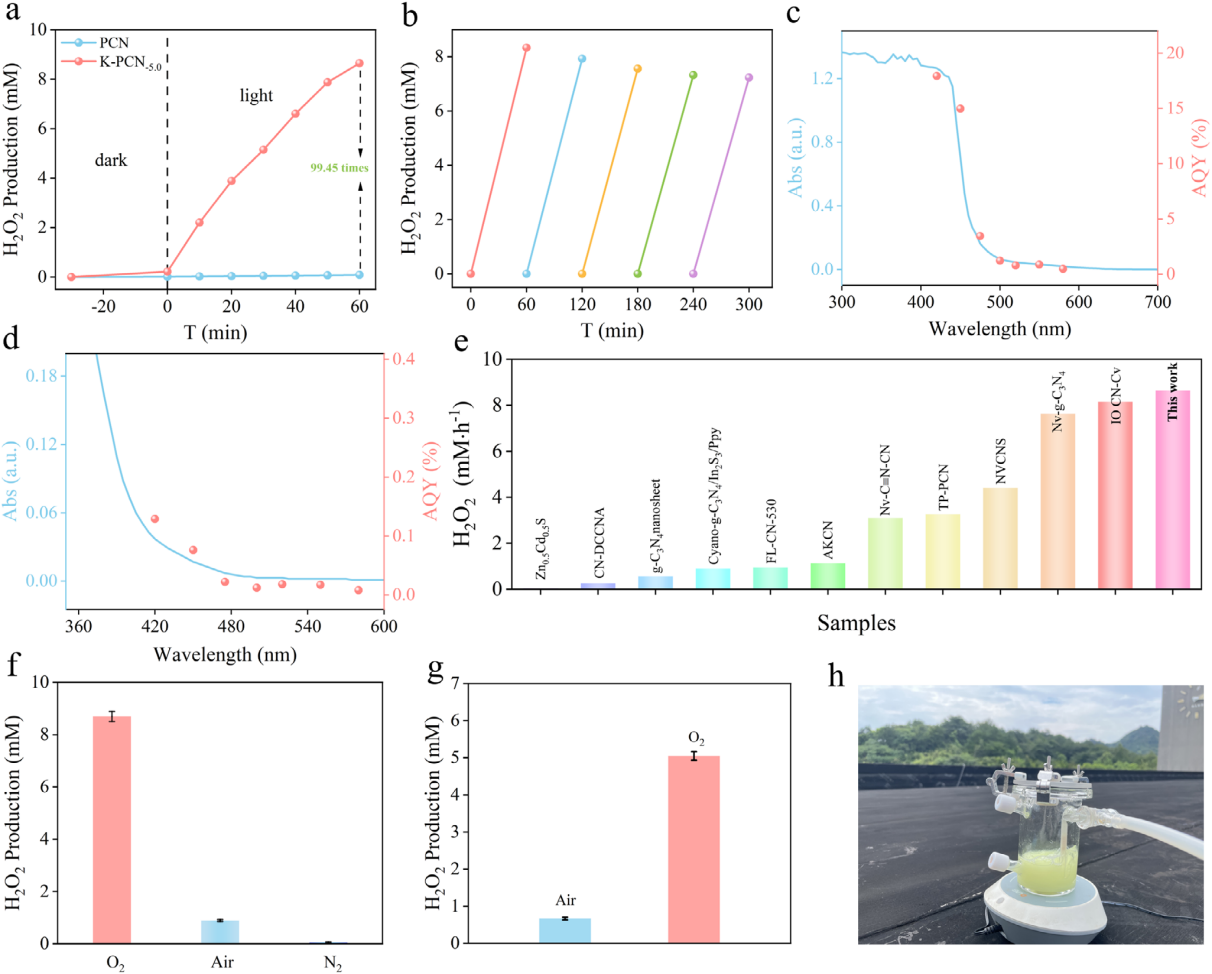
a) Photocatalytic H_2_O_2_ generation efficiency of PCN and K‐PCN_‐5.0_; b) Cycling stability of K‐PCN_‐5.0_; AQY of c) K‐PCN_‐5.0_ and d) PCN under different wavelengths; e) Comparison with other photocatalysts from recent works for H_2_O_2_ production photocatalysts; f) Photocatalytic H_2_O_2_ production performance of K‐PCN_‐5.0_ under different reaction gases and Xe lamp irradiation; g) Photocatalytic H_2_O_2_ production performance of K‐PCN_‐5.0_ under different reaction and sunlight irradiation; h) Setup for photocatalytic H_2_O_2_ production with by K‐PCN_‐5.0_ under sunlight irradiation.

The H_2_O_2_ production capacity of both the PCN and K‐PCN_‐x_ via O_2_ reduction was evaluated under visible light. The concentration of H_2_O_2_ generated in the photocatalytic system was determined using a standard calibration curve for H_2_O_2_ (Figure S13, Supporting Information). Little H_2_O_2_ can be detected in the absence of a photocatalyst, whereas 26.3 µm of H_2_O_2_ was produced within 1 h when K‐PCN was used as the catalyst in pure deionized water (Figure S14a,b, Supporting Information). The influence of various sacrificial agents, including glycerol, methanol, ethylene glycol, ethanol, and isopropanol, on photocatalytic H_2_O_2_ production was investigated using the K‐PCN_‐5.0_ catalyst. As shown in Figure S15a (Supporting Information), the greatest H_2_O_2_ generation efficiency of 8647.78 µM·h^−1^ was obtained when using isopropanol as the trapping agent. The amount of H_2_O_2_ production is 1507.7, 8647.78, and 4149.73 µM for K‐PCN_‐2.5_, K‐PCN_‐5.0_, and K‐PCN_‐7.5_, respectively (Figure S15b, Supporting Information), while the pristine PCN can only produce 86.96 µM under identical conditions. The H_2_O_2_ production rate of K‐PCN_‐5.0_ is 99.45 times higher than PCN (**Figure** **4**a), demonstrating the effective improvement of photocatalytic performance by K‐decorated carbon nitride.

The influence of photocatalyst preparation temperature of photocatalyst on the photocatalytic capability of K‐PCN_‐5.0_ was also investigated (Figure S15c, Supporting Information). Photocatalytic activity increased with temperature, reaching an optimal point at 570 °C before decreasing, likely owing to structural degradation at higher temperatures.^[^ ^47^ ^]^ K‐PCN_‐5.0_ achieved a high H_2_O_2_ production rate under 80 mL/min aeration conditions at pH 11.0 (Figure S15d, Supporting Information) for a 0.10 g dosage (Figure S15e,f, Supporting Information). Moreover, the influence of sacrificial‐agent content on the H_2_O_2_ production rate was examined. As illustrated in Figure S16a (Supporting Information), the production efficiency of hydrogen peroxide increases as the amount of sacrificial agent rises. The practical utility of K‐PCN_‐5.0_ was further evaluated by assessing its catalytic stability (Figure 4b). The catalytic activity of K‐PCN_‐5.0_ can be observed to remain nearly unchanged after five consecutive cycles, demonstrating its excellent stability. In addition, the photocatalytic H_2_O_2_ production of K‐PCN_‐5.0_ in real‐water matrices was examined. As illustrated in Figure S16b (Supporting Information), the H_2_O_2_ production rate in tap water or lake water is lower than that in ultrapure water, primarily because the ions present in actual water inhibit H_2_O_2_ generation, and the formation of precipitates blocks the reactive active sites. Furthermore, the K‐PCN_‐5.0_ catalyst was recovered after the reaction to assess any potential changes in its chemical structure. SEM (Figure S17, Supporting Information) and XRD (Figure S18, Supporting Information) analyses demonstrated that the used K‐PCN_‐5.0_ retained the same structural features as the fresh catalyst, confirming its structural integrity and strong resistance to photocorrosion. The AQY of K‐PCN_‐5.0_ and PCN were measured under different wavelength ranges to evaluate their light utilization efficiency. As shown in Figure 4c and K‐PCN_‐5.0_ achieves an AQY of 17.92% at 420 nm (Table S3, Supporting Information), far surpassing that of pristine PCN (0.129%; Figure 4d; Table S4, Supporting Information). Moreover, compared with other CN‐based heterogeneous systems (Table S5, Supporting Information), the K‐PCN_‐5.0_ system exhibits significantly higher efficiency in photocatalytic H_2_O_2_ production (Figure 4e).

To further investigate the pathway of H_2_O_2_ production by K‐PCN_‐5.0_, photocatalytic performance tests were conducted under different gas atmospheres (Figure 4f). The H_2_O_2_ production rate of K‐PCN_‐5.0_ reached 8647.78 µM·h^−1^ in an oxygen‐saturated solution (Figure S19a, Supporting Information), but decreased significantly to 889.61 µM·h^−1^ under air‐saturated conditions. Notably, when a nitrogen‐saturated solution was used (Figure 4f; Figure S19b, Supporting Information), the rate decreased further to 54.74 µM·h^−1^. Collectively, these results suggest that H_2_O_2_ production over K‐PCN_‐5.0_ primarily follows the two‐electron oxygen reduction reaction (2e^−^ ORR) pathway, while water oxidation contributes only marginally. To better simulate practical conditions, a comparative test was conducted using K‐PCN_‐5.0_ in an air‐saturated solution under natural sunlight (Figure 4g,h). The H_2_O_2_ production rate can be observed to reach 667.11 µM·h^−1^—≈78% that observed under 300 W xenon lamp irradiation (848.82 µM·h^−1^)—demonstrating that the abundant active sites^[^ ^48^ ^]^ in K‐PCN_‐5.0_ exhibit strong sunlight utilization capability. More importantly, the H_2_O_2_ production rate in an oxygen‐saturated solution (5000 µm·h^−1^) is significantly higher than that in air atmosphere (667.11 µm·h^−1^) under irradiation of sunlight (Figure 4g). It is well established that H_2_O_2_ undergoes decomposition during the photocatalytic H_2_O_2_ production process. Therefore, the H_2_O_2_ decomposition experiment was conducted under identical conditions. As shown in Figure S20 (Supporting Information), H_2_O_2_ decomposed by 7.73% within 1 h in the PCN system under continuous N_2_ purging, whereas a decomposition of 48.84% occurred in the K‐PCN‐5.0 system. This directly confirms that K‐PCN_‐5.0_ has a significantly high net efficiency for H_2_O_2_ generation. Photocatalytic H_2_O_2_ production using the K‐PCN_‐5.0_ catalyst was conducted to demonstrate its practical application potential. The generated H_2_O_2_ was subsequently applied for dye wastewater decolorization (Figure S21a, Supporting Information). The photocatalytically produced H_2_O_2_ was utilized for dye wastewater decolorization using Fenton‐based treatment. Remarkably, even at a low concentration (≈8.65 mm), the H_2_O_2_ effectively degraded methylene blue (MB) and Rhodamine B (RhB) (Figure S21b,c, Supporting Information), highlighting the potential of this system for practical water treatment applications.

### 2.3 Mechanism of Photocatalytic H_2_O_2_ Generation

**Figure 5:**
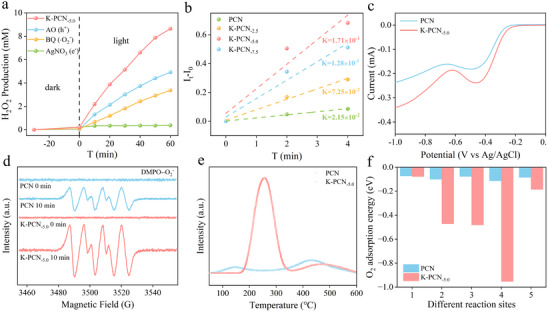
a) Photocatalytic activity of K‐PCN_‐5.0_ on H_2_O_2_ production with different scavengers; b) Relative •O_2_^−^ production rate via NBT trapping experiments; c) LSV curves of PCN and K‐PCN_‐5.0_ measured via RRDE analysis at 2,500 rpm; d) Signal of •O_2_‐DMPO for PCN and K‐PCN_‐5.0_; e) O_2_‐TPD of PCN and K‐PCN_‐5.0_; f) Free energy of oxygen adsorption (*E*_ads_) at different adsorption sites of PCN and K‐PCN_‐5.0_ by DFT.

A set of controlled experiments was performed to elucidate the mechanism of H_2_O_2_ photosynthesis on K‐PCN_‐5.0_. As depicted in **Figure** **5**a, little H_2_O_2_ production with AgNO_3_ (electron scavenger), along with the sharp decline in H_2_O_2_ generation when benzoquinone (BQ, a •O_2_^−^ trapping agent) was introduced, indicates that H_2_O_2_ is formed via an indirect two‐electron reduction pathway, with •O_2_^−^ acting as a key intermediate (O_2_ + e^−^→ •O_2_^−^; •O_2_^−^ + e^−^ + 2H^+^ → H_2_O_2_). Nitroblue Tetrazolium (NBT) trapping tests were performed to quantify the generation of superoxide radicals (•O_2_^−^). NBT selectively reacted with •O_2_^−^ to generate a blue complex precipitate, the concentration change of which was monitored by measuring the absorbance at 259 nm using a UV–vis spectrophotometer.^[^ ^36^ ^]^ The variation in NBT concentration over irradiation time for different photocatalyst systems is shown in Figure S22 (Supporting Information). In the K‐PCN system, the NBT concentration decreased sharply within 4 min, demonstrating rapid •O_2_^−^ generation. In contrast, the PCN system exhibited a negligible change in NBT concentration, suggesting sluggish oxygen reduction kinetics. The kinetics of •O_2_^−^ generation were estimated using the equation: *k* = ‐d*C*/dt, where *k* is the reaction rate and d*C* represents the change in •O_2_^−^ concentration over time (*dt*), as indicated by the decrease in NBT absorbance at 259 nm. The rate of •O_2_^−^ generation by K‐PCN was found to be nearly 11 times higher than that by PCN (Figure 5b). In addition, a robust linear correlation was found between the rates of H_2_O_2_ and •O_2_^−^ generation (Figure S23, Supporting Information), providing further evidence that H_2_O_2_ is generated via a two‐electron oxygen reduction reaction (2e^−^ ORR) pathway.

The generation of H_2_O_2_ during photocatalysis suggests the involvement of an oxygen reduction reaction (ORR). To investigate this, rotating ring‐disk electrode (RRDE) polarization curves were recorded for the samples at various rotation speeds (Figure S24, Supporting Information). A sharp increase in reduction current was revealed at the disk electrode between −0.2 and −0.4 V, corresponding to the rapid reduction of O_2_ to H_2_O_2_. Notably, at a rotation speed of 2500 rpm (Figure 5c), K‐PCN_‐5.0_ exhibits the highest reduction current, significantly exceeding that of pristine PCN. This result indicates that K‐PCN_‐5.0_ possesses superior oxygen reduction capability.^[^ ^49^ ^]^ The electron transfer number was also determined using RRDE analysis. As shown in Figure S25 and Table S6 (Supporting Information) (RED data at −0.4 V), the electron transfer number for K‐PCN_‐5.0_ was closer to 2 than that of pristine PCN, indicating that the ORR involved in H_2_O_2_ production by K‐PCN_‐5.0_ follows a two‐electron pathway.

The reactive radical species involved in the reaction procedure were identified using electron spin resonance (ESR; Figure 5d), and no DMPO‐•O_2_^−^ signal can be observed under dark conditions (0 min), however, after 10 min of light irradiation, a clear •O_2_^−^ signal emerges. The signal intensity of K‐PCN_‐5.0_ is notably stronger than that of PCN, which is consistent with the findings of the radical trapping experiments. Similarly, distinct signals corresponding to ^1^O_2_ (Figure S26a, Supporting Information) and •OH (Figure S26b, Supporting Information) were also observed. These findings confirm the involvement of •O_2_^−^ and ^1^O_2_ as key intermediates in the photocatalytic H_2_O_2_ production procedure, as well as the formation of •OH from subsequent H_2_O_2_ decomposition.^[^ ^50^ ^]^ As shown in Figure 5e and K‐PCN_‐5.0_ exhibits significantly stronger O_2_‐ temperature‐programmed desorption (TPD) signals than pristine PCN, demonstrating the markedly enhanced O_2_ adsorption capacity of PCN owing to the incorporation of K^+^. This improved adsorption was directly correlated with the subsequent oxygen reduction process. The possible O_2_ adsorption sites on PCN and K‐PCN_‐5.0_ were determined via DFT calculations. The results revealed that in the K‐PCN_‐5.0_ model, the ─C≡N group exhibits the lowest binding energy of −0.95 eV, followed by ─OH with binding energies of −0.47 and −0.48 eV (Figure 5f and S27, Supporting Information). This indicates that the ─C≡N and ─OH have stronger adsorption affinity for O_2_ than other sites. This enhanced adsorption is attributed to the electron‐withdrawing properties of these groups, further promoting the local electron density and thereby strengthening O_2_ binding.

**Figure 6:**
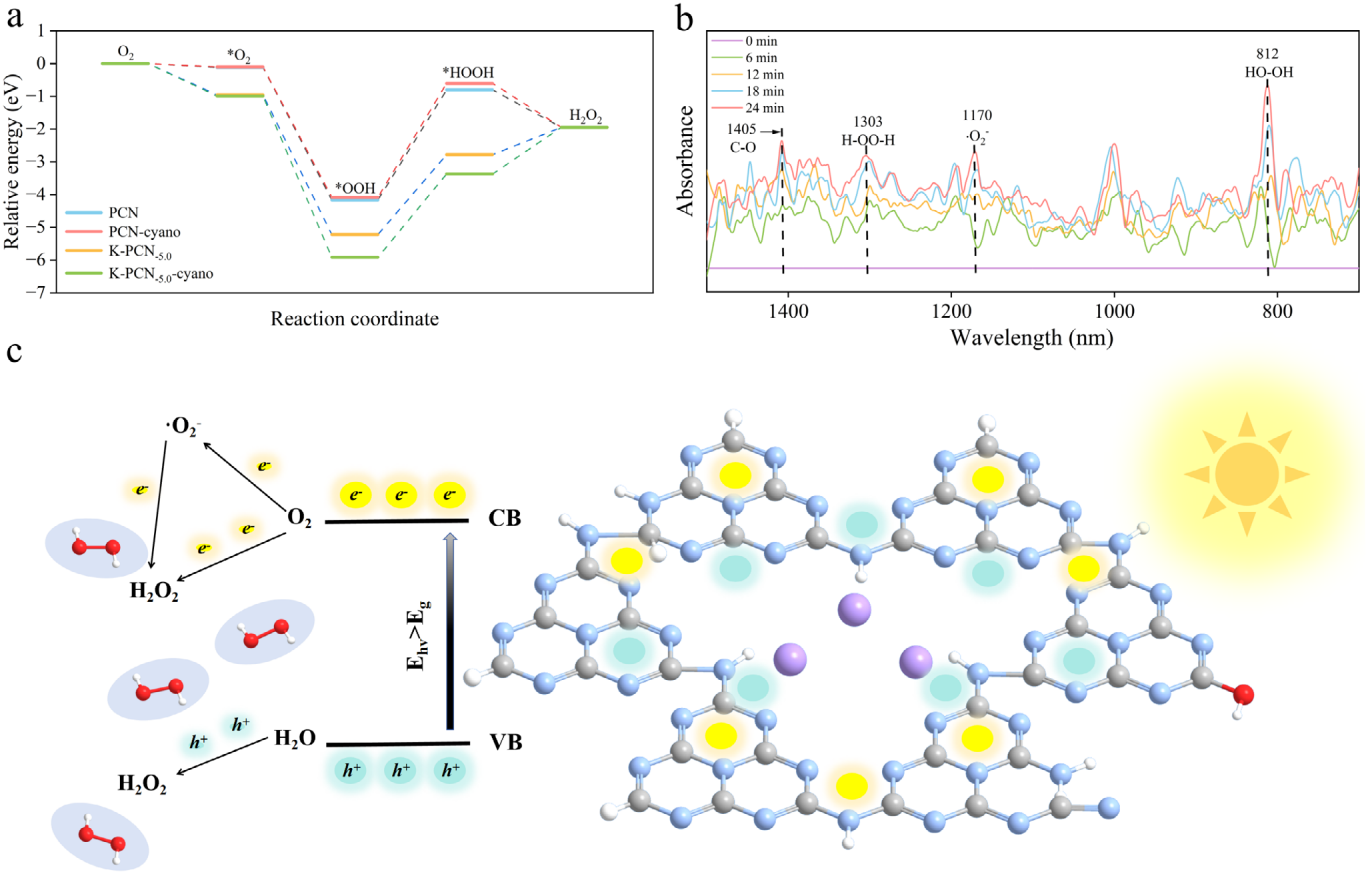
a) Oxygen adsorption sites of K‐PCN_‐5.0_ and PCN; b) In situ FTIR spectra of K‐PCN_‐5.0_ photocatalytic production of H_2_O_2_ under 300 W xenon lamp irradiation and O_2_ fluxing; c) Mechanism of H_2_O_2_ generation by K‐PCN photocatalysis.

To elucidate the pathway for efficient H_2_O_2_ production via K‐PCN_‐5.0_ photocatalysis, the relative energies along the ^*^O_2_ → ^*^OOH → ^*^H_2_O_2_ reaction pathway (where ^*^ denotes adsorbed species) were analyzed using DFT calculations (**Figure** **6**a). On the surface of pristine PCN, ^*^O_2_ activated with difficulty and reduced to ^*^OOH. However, the introduction of K⁺ considerably decreased the energy barrier for ^*^O_2_ → ^*^OOH, thereby facilitating H_2_O_2_ formation. Notably, K‐PCN_‐5.0_ possessed a larger specific surface area than pristine PCN, offering additional active sites for O_2_ adsorption (Figure S28, Supporting Information). This observation is consistent with the O_2_ temperature‐programmed desorption (TPD) results demonstrated in Figure 5e. The mechanism of H_2_O_2_ production by K‐PCN_‐5.0_ photocatalysis was further investigated using in situ FTIR spectroscopy (Figure 6b). To ensure consistency, the test conditions were the same as those used in the photocatalytic H_2_O_2_ production experiments. The absorption peak at 1170 cm^−1^ was assigned to •O_2_^−^ and enhanced by prolonged light irradiation, indicating that the adsorbed O_2_ on K‐PCN_‐5.0_ is progressively reduced to •O_2_^−^ as an intermediate species.^[^ ^6^ ^]^ The absorption peaks at 812 and 1303 cm^−1^ corresponded to the O─O stretching mode of H_2_O_2_ and the bending vibration of the O─H bonds, respectively, which also increased with irradiation time. This suggests that the adsorbed O_2_ is continuously reduced to H_2_O_2_ during the photocatalytic process.^[^ ^51^ ^]^ In addition, the peak at 1405 cm^−1^ was ascribed to C─O, indicating the gradual formation of ─OH groups during the photocatalytic process.^[^ ^37^ ^]^ These results demonstrate that K‐PCN_‐5.0_ exhibits efficient and stable photocatalytic H_2_O_2_ production. The proposed mechanism for the process was exemplified (Figure 6c). Doping PCN with K^+^ increases the number of reactive sites and reduces the interlayer spacing, thereby promoting O_2_ adsorption and exciton dissociation. Additionally, K⁺ doping narrows the catalyst's band gap, improves the division rate of photoinduced carriers, and shifts the valence band (VB) and conduction band (CB) more positively, thus facilitating O_2_ reduction. Upon photoexcitation, electrons are promoted from VB to CB, generating holes in the VB. The adsorbed O_2_ is then reduced to •O_2_^−^, which is further reduced to H_2_O_2_ in the CB, whereas water is oxidized to H_2_O_2_ in the VB.

## 3 Conclusion

In summary, we developed a straightforward approach for preparing K⁺‐doped PCN photocatalysts that effectively enhances H_2_O_2_ production. The interlayer K⁺ ions acted as bridges, improved charge transfer between PCN layers, while abundant surface ─C≡N and ─OH groups promoted intralayer charge movement and increased active sites for oxygen reduction. The resulting K‐PCN_‐5.0_ exhibited superior photocatalytic performance and achieved a high H_2_O_2_ generation rate both in situ and under sunlight, outperforming most modified PCN catalysts. Mechanistic studies confirmed that H_2_O_2_ is formed via a two‐step single‐electron oxygen reduction pathway. Furthermore, K‐PCN_‐5.0_ demonstrated excellent stability and reusability, underscoring its practical potential. This study delivers vital insights into intercalation engineering strategies for the synthesis of effective photocatalytic systems and clarifies the underlying mechanisms of photocatalytic H_2_O_2_ generation.

## Conflict of Interest

The authors declare no conflict of interest.

## Supporting information



Supporting Information

## Data Availability

The datasets generated or analyzed during the current study are available from the corresponding author upon reasonable request.
