# Supplementary material for: Electronic Modulation of K⁺‐Intercalated Polymeric Carbon Nitride via ─C≡N/─OH Functionalization for Efficient Photocatalytic H2O2 Production
Source: Adv Sci (Weinh). 2025 Dec 7;13(11):e21125. doi: 10.1002/advs.202521125 (PMC12931227; doi:10.1002/advs.202521125)
Supplement: Supplementary file 1 — Supporting Information [file ADVS-13-e21125-s001.docx]

**Supporting Information**

Electronic Modulation of K⁺-Intercalated Polymeric Carbon Nitride *via* ─C≡N/─OH Functionalization for Efficient Photocatalytic H_2_O_2_ Production

*Qingquan Xue, Shuang Liu, Ye Yang, Jiaxiang Li, Fan Tang, Shengdao Shan, Xiaofeng Shen,^*^ Hua Pan^*^ and Chi He^*^*

**Table of Contents**

[Experimental procedures S4](#_Toc214524228)

[Chemicals and reagents S4](#_Toc214524229)

[Preparation of photocatalysts S4](#_Toc214524230)

[Characterizations S4](#_Toc214524231)

[Electrochemical testing S5](#_Toc214524232)

[Evaluation of photocatalytic activity of H_2_O_2_ production S5](#_Toc214524233)

[In-situ infrared procedure for monitoring H_2_O_2_ production with K-PCN_-5.0_ S6](#_Toc214524234)

[Density functional theory (DFT) calculations S6](#_Toc214524235)

[Statistical Analysis S7](#_Toc214524236)

[Figure S1. Planar structure of PCN and planar and spatial structure of K-PCN. S8](#_Toc214524237)

[Figure S2. SEM images of (a) PCN, (b) K-PCN_-2.5_, (c) K-PCN_-5.0_, and (d) K-PCN_-7.5_; TEM images of (e) K-PCN_-5.0_ and (f) PCN. S8](#_Toc214524238)

[Figure S3. Element distribiution image of K-PCN_-5.0_. S9](#_Toc214524239)

[Figure S4. Photographs of samples freely settling in ultrapure water for 7 days. From left to right are blank, PCN, and K-PCN_-5.0_. S9](#_Toc214524240)

[Figure S5. (a) Hydrodynamic size distribution and (b) zeta potential of K-PCN_-5.0_ and PCN. S9](#_Toc214524241)

[Figure S6. (a) XRD and (b) FTIR of prepared samples. S10](#_Toc214524242)

[Figure S7. (a) XPS survey and (b) XPS spectra valence band (VB) positions of PCN and K-PCN_-5.0_; High-resolution XPS spectra of (c) C 1*s*, (d) N 1*s*, (e) O 1*s*, and (i) K 2*p* of PCN and K-PCN_-5.0_. S10](#_Toc214524243)

[Figure S8. Time-resolved PL spectra of prepared samples. S11](#_Toc214524244)

[Figure S9. EIS and transient photocurrent responses of PCN and K-PCN. S11](#_Toc214524245)

[Figure S10. (a) UV-Vis DRS and (b) band-gap analysis diagram of PCN and K-PCN. S12](#_Toc214524246)

[Figure S11. Mott-Schottky curves of (a) PCN, (b) K-PCN_-2.5_, (c) K-PCN_-5.0_, and (d) K-PCN_-7.5_. S12](#_Toc214524247)

[Figure S12. Electrostatic potential surface distribution of PCN and K-PCN_-5.0_. S13](#_Toc214524248)

[Figure S13. Standard curves of (a) low and (b) high concentrations of H_2_O_2_ calibrated by the iodination method. S13](#_Toc214524249)

[Figure S14. (a) Photocatalytic experiments with K-PCN in the absence of a sacrificial agent; (b) Photocatalytic blank experiment without catalyst. S13](#_Toc214524250)

[Figure S15. Photocatalytic H_2_O_2_ production performance of K-PCN under different (a) sacrificial agents, (b) alkali metal doping ratios, (c) preparation temperatures, (d) solution pH values, (e) O_2_ fluxes, and (f) catalyst qualities. S14](#_Toc214524251)

[Figure S17. SEM image of K-PCN_-5.0_ after photocatalytic reaction. S15](#_Toc214524252)

[Figure S18. XRD comparison of samples before and after photocatalytic reaction. S15](#_Toc214524253)

[Figure S19. Photocatalytic experiments when (a) O_2_ and (b) N_2_ were introduced. S16](#_Toc214524254)

[Figure S20. H_2_O_2_ decomposition properties of different samples in N_2_ atmosphere. S16](#_Toc214524255)

[Figure S21. (a) K-PCN_-5.0_ photocatalyst was used for photocatalytic reaction to generate H_2_O_2_ and the generated H_2_O_2_ was used for decolorization of dye wastewater. Full wavelength scanning of (b) MB and (c) RhB solutions. S17](#_Toc214524256)

[Figure S22. The absorption intensity of NBT at 259 nm in (a) PCN, (b) K-PCN_-2.5_, (c) K-PCN_-5.0_, and (d) K-PCN_-7.5_ reaction solutions. S18](#_Toc214524257)

[Figure S23. Relationship between H_2_O_2_ generation rate and the •O_2_^-^ intermediate. S18](#_Toc214524258)

[Figure S24. RRDE polarization curves of (a) PCN and (b) K-PCN_-5.0_ in O_2_-saturated KOH (0.1 M) solution at different rotational speeds. S19](#_Toc214524259)

[Figure S25. Electron transfer numbers of (a) K-PCN_-5.0_ and (b) PCN fitted by the K-L equation. S19](#_Toc214524260)

[Figure S26.The signal of (a) ^1^O_2_-TEMP and (b) •OH-DMPO. S19](#_Toc214524261)

[Figure S27. Calculation of oxygen adsorption sites of simulated (a) PCN and (b) K-PCN_-5.0_. S20](#_Toc214524262)

[Figure S28. (a) The nitrogen adsorption-desorption isotherm and specific surface area (insert) of prepared samples. (b) Pore size distribution of prepared samples. S20](#_Toc214524263)

[Table. S1. The values of TRPL lifetime τ_i_ (E_λ_ = 360 nm) and corresponding constant A_i_. S21](#_Toc214524264)

[Table. S2. Conversion of reference electrode potentials for samples. S21](#_Toc214524265)

[Table. S3. Parameters for calculating AQY for H_2_O_2_ production by K-PCN_-5.0_ photocatalysis. S22](#_Toc214524266)

[Table. S4. Parameters for calculating AQY for H_2_O_2_ production by PCN photocatalysis. S22](#_Toc214524267)

[Table. S5. Comparison of the performance of photocatalytic H_2_O_2_ production in the reported literature. S23](#_Toc214524268)

[Table. S6. The number of transferred electrons for K-PCN_-5.0_ and PCN fitted by the K-L equation. S24](#_Toc214524269)

[**References** S25](#_Toc214524270)

# Experimental procedures

# Chemicals and reagents

Melamine (C_3_H_6_N_6_, 99% , Aladdin Reagent), potassium chloride (KCl, AR, Aladdin Reagent), hydrochloric acid (HCl, AR, Ling Feng Reagent), sodium hydroxide (NaOH, AR, Aladdin Reagent), silver nitrate (AgNO_3_, AR, Aladdin Reagent), P-benzoquinone (PBQ, 99%, Aladdin Reagent), ammonium oxalate (AO, AR, Aladdin Reagent), isopropyl alcohol (IPA, AR, Aladdin Reagent), methanol (CH_3_OH, AR, Aladdin Reagent), ethanol (C_2_H_5_OH, AR, Aladdin Reagent), ethylene glycol (C_2_H_6_O_2_, AR, Aladdin Reagent), glycerol (C_3_H_8_O_3_, AR, Aladdin Reagent), The nitroblue tetrazolium (NBT, 98%, Aladdin Reagent), tetracycline hydrochloride (TC, 96%, Aladdin Reagent), potassium iodide (KI, AR, Aladdin Reagent), potassium hydrogen phthalate (C_8_H_5_O_4_K, 99.8%, Aladdin Reagent) were used directly without further purification.

# Preparation of photocatalysts

**Preparation of PCN:** The original PCN was synthesized by using a traditional thermal polycondensation method. Melamine was placed in a porcelain boat with a lid. Then it was heated up to 425 ℃ under N_2_ atmosphere at a heating rate of 2 ℃/min and kept for 4 h to obtain Melem which was further put into a porcelain boat with a lid, under N_2_ atmosphere, and heated to 550 ℃ under N_2_ atmosphere and kept for 4 h with a heating rate of 2 ℃/min to obtain a light-yellow solid.

**Preparation of K-PCN:** 1.0 g of Melem and a certain amount of KCl (2.5, 5.0, and 7.5 g) were placed into a mortar and thoroughly grinding to obtain a solid mixture. The mixture was then placed in a porcelain boat with a lid and heated to 570 ℃ for 4 h under N_2_ atmosphere at a heating rate of 2 ℃/min to obtain a yellow solid. After cool down the room temperature, the product was stirred with deionized water to fully remove the residue salts, and finally filtered to obtain carbon nitride polymer (K-PCN_-X_, x = 2.5, 5.0, and 7.5, representing the amount of potassium salt) yellow powder.

# Characterizations

The morphology of prepared samples was observed by field emission scanning electron microscopy (FE-SEM, Regulus8100, Japan) and transmission electron microscopy (TEM, Talos F200, USA). X-ray diffraction patterns of prepared samples were measured using an X-ray diffractometer (XRD-6100, Japan). Fourier transform infrared spectra (FT-IR) of prepared samples were measured using an infrared spectrometer (Nicolet is50, USA). Sample ^13^C high-resolution solid-state NMR analysis was performed using a solid-state nuclear magnetic resonance spectrometer (Bruker 400 M, Germany). The X-ray photoelectron spectra (XPS) of prepared samples were measured using an X-ray photoelectron spectrometer (Escalab 250xi, USA).The specific surface area and pore size distribution of prepared samples were measured using a specific surface area and pore size analyzer (BET, ASAP2460, USA). The absorption spectra of prepared samples were analyzed using a UV-Vis spectrophotometer (UV3600, Japan). Room temperature photoluminescence (PL) spectra were measured by a fluorescence spectrometer (FLS1000, UK). Room temperature time-resolved fluorescence lifetimes (TR-PL) were performed on a fluorescence spectrophotometer (FLS1000, UK) with an excitation wavelength of 360 nm. Electrochemical measurements were performed on the samples using an electrochemical workstation (PGSTAT204, The Netherlands). The main active substances in the photocatalytic process were measured with a paramagnetic resonance spectrometer (ESR, Bruker A300, Germany). The oxygen desorption characteristics of the samples were measured using a fully automated adsorption meter (TP-5080-B, China). The zeta potential of the material and the relationship between the particle size distribution and the intensity distribution were measured by a nanoparticle size analyzer (Malvern Zetasizer Nano ZS90, UK). The surface potential of the samples was measured using a Kelvin probe microscope (KPFM, Dimension Icon, Germany). The hydrodynamic diameter of the samples was measured using a dynamic light scatterometer (DLS, Malvern Zetasizer Nano ZS90, UK). The amount of H_2_O_2_ was measured using a UV-Vis spectrophotometer (DR6000, USA).

# Electrochemical testing

Specifically, 450 μL of ethanol solution and 50 μL of naphthol solution (used for bonding and enhancing detection sensitivity) were accurately pipetted into a 5 mL centrifuge tube. Then the K-PCN_-5.0_ (50 mg) was added to abovementioned mixture and sonicated for 30 min. The prepared suspension was uniformly drop-cast onto a 1 × 1 cm^2^ FTO glass slide, followed by drying under infrared irradiation for 10 minutes. The electrochemical setup employed a platinum counter electrode and an Ag/AgCl reference electrode, with 0.2 M Na_2_SO_4_ used as the electrolyte.

# Evaluation of photocatalytic activity of H_2_O_2_ production

Specifically, 100 mL of solvent (containing 90% ultrapure water and 10% sacrificial agent) was added to the photocatalytic reactor. The inlet of the reactor and the oxygen cylinder were connected through a flexible hose, and the outlet was vented into the water. Afterwards, the magnetic stirrer was turned on and the oxygen aeration was adjusted at 80 mL/min until stabilization, and O_2_ saturation was reached in the reactor after 30 min. Specifically, to emphasize the effect of xenon light, we kept the reactor in a light-free state during this 30 min (using an opaque container to cover the reactor). At the end of the above process, 1.5 mL of the reaction solution (0 min) was taken and the catalyst was filtered off using a 0.22 μm filter membrane. The xenon lamp was turned on for irradiation, after which the reaction solution was taken every 10 min, resulting in 7 portions of reaction solution (0, 10, 20, 30, 40, 50, and 60 min). To the obtained reaction solution, 0.75 mL of potassium iodide solution (0.4 M) and 0.75 mL of potassium hydrogen phthalate solution (0.1 M) were added, waited for 10 min and then the absorbance was measured at 350 nm using UV-Vis spectrophotometer (For the blank control, ultra-pure water was used to replace the reaction solution, and 0.75 mL of potassium iodide solution together with 0.75 mL of potassium hydrogen phthalate solution was added. The absorbance of the blank was set to zero.).

In addition, the actual solar H_2_O_2_ generation experiment was conducted at the Experimental Building of Zhejiang University of Science and Technology, West Lake District, Hangzhou City, Zhejiang Province, China (30.133°N 120.123°E) at 1:00 p.m. on August 1, 2025, with a solar irradiance intensity of 24.05 mW/cm^2^.

# In-situ infrared procedure for monitoring H_2_O_2_ production with K-PCN_-5.0_

Specifically, 10 mg of dried sample was uniformly coated on the IR window sheet to form a uniformly distributed film and placed inside the chamber under dark conditions with O_2_ (oxygen cylinder) and water vapor (bubbler) for 30 min and the background spectrum was collected. The O_2_ and water vapor were kept on, the cavity was illuminated with a xenon lamp, and the scanning mode was turned on (spectral resolution of 8 cm^-1^). Data were collected for differential spectral processing (all light data minus dark data).

# Density functional theory (DFT) calculations

All optimizations were done with periodic spin-polarized density functional theory (DFT) implemented in the ViennaAb initio Simulation Package (VASP 5.4.4). The projector augmented wave (PAW) method and the Perdew-BurkeErnzerhof (PBE) method were used to describe the exchange-correlation. The cutoff energy of the plane-wave basis sets was set to 450 eV, and the k-point meshes of Γ based on the Monkhorst-Pack scheme at the Γ point from the Brillouin zone were used in all models. The geometry optimizations were done with a convergence criterion of 10^-5^ eV for total energy, and atoms were permitted to relax until the maximum force was less than 0.05 eV/Å.

# Statistical Analysis

The photocatalytic H_2_O_2_ production using PCN and K-PCN_-5.0_ as catalysts was determined based on the standard-curve method (Figure S13). In addition, to guarantee the reproducibility and accuracy of the data, the photocatalytic H_2_O_2_ production of K-PCN_-5.0_ under different atmospheres (air, N_2_, and O_2_-saturated) and under either Xe-lamp irradiation or natural sunlight was evaluated three times (n = 3). And the statistical analysis for photocatalytic H_2_O_2_ production were calculated using standard functions in Microsoft Excel software.


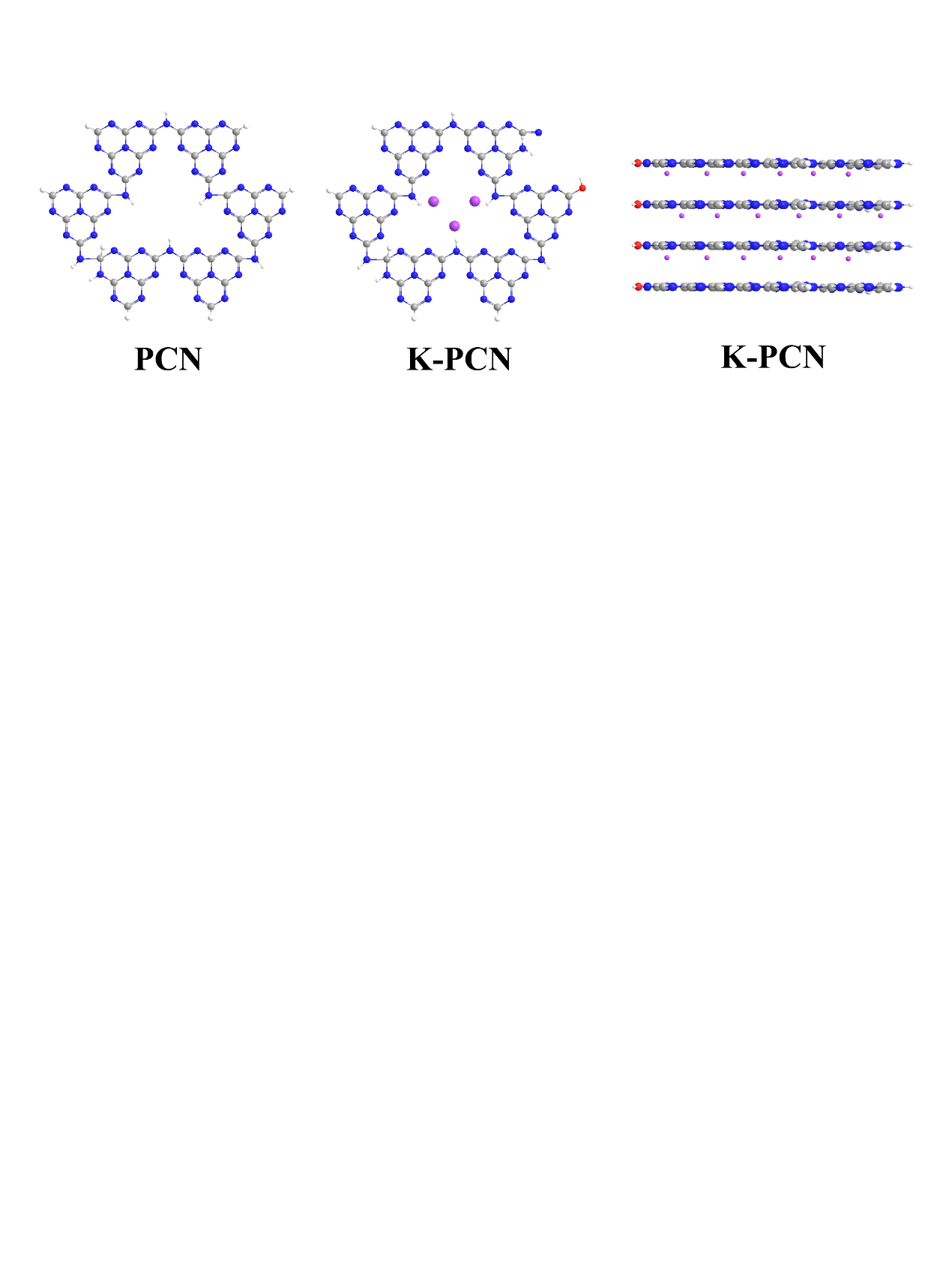


# Figure S1. Planar structure of PCN and planar and spatial structure of K-PCN.


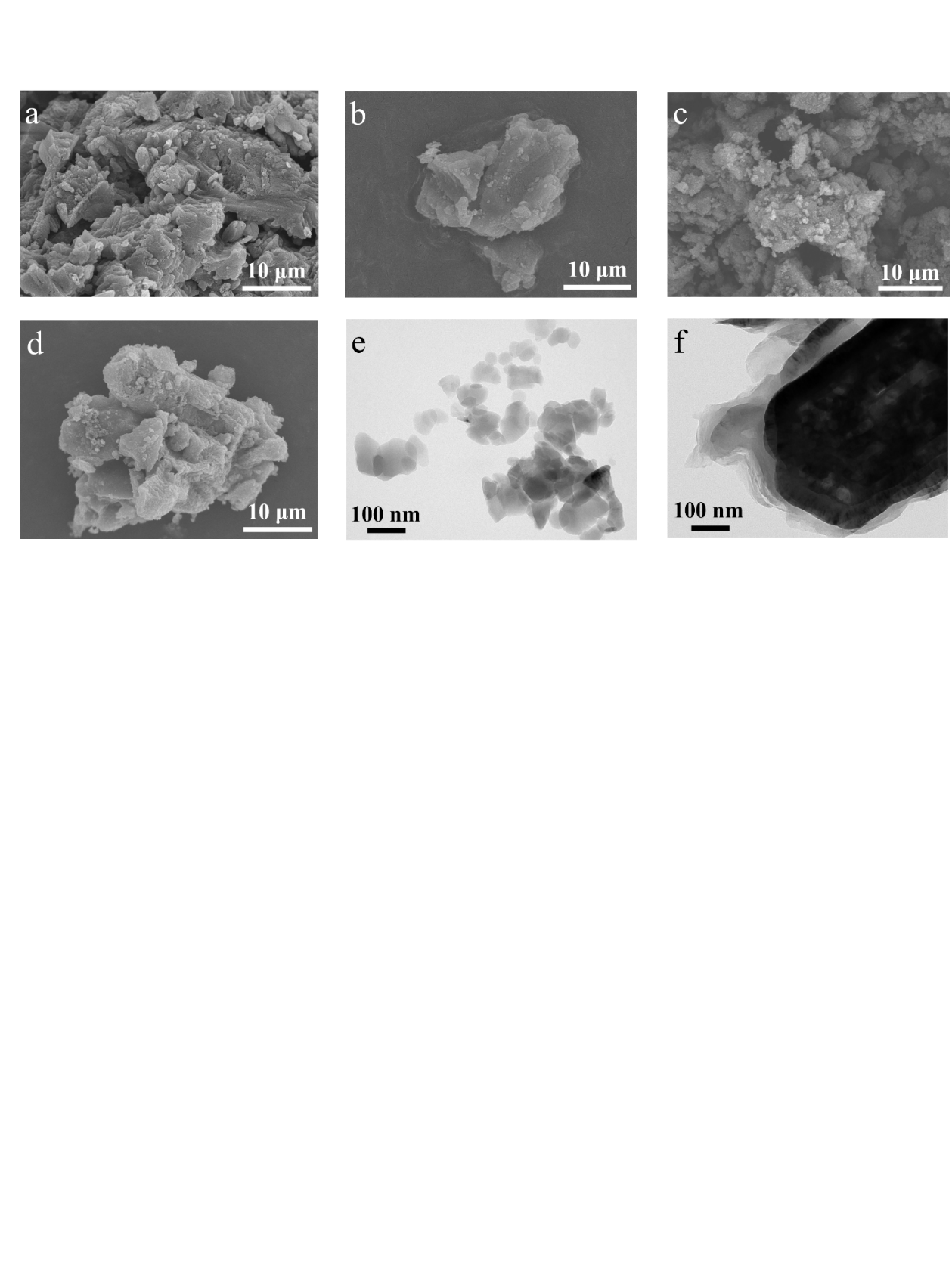


# Figure S2. SEM images of (a) PCN, (b) K-PCN_-2.5_, (c) K-PCN_-5.0_, and (d) K-PCN_-7.5_; TEM images of (e) K-PCN_-5.0_ and (f) PCN.


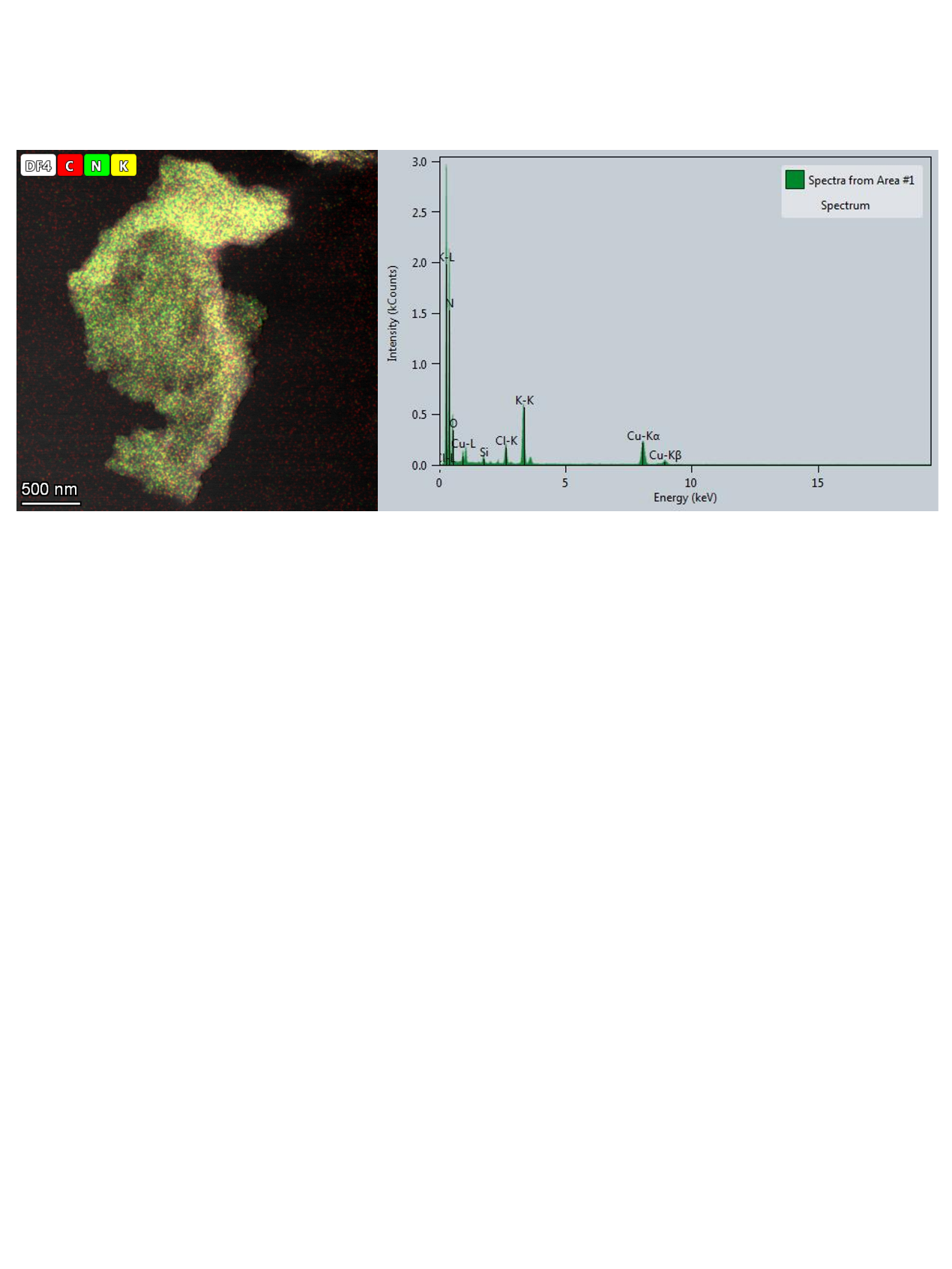


# Figure S3. Element distribiution image of K-PCN_-5.0_.


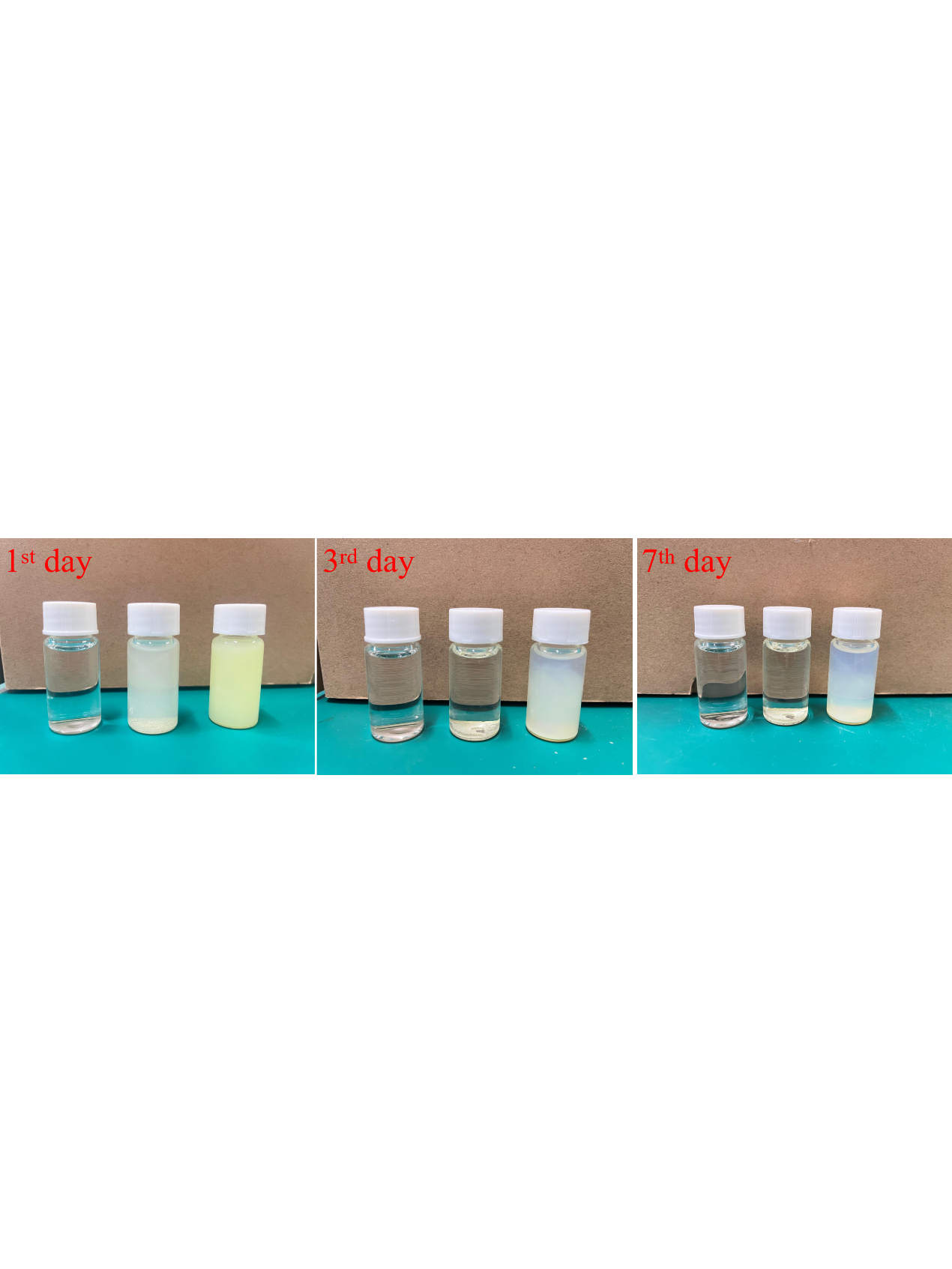


# Figure S4. Photographs of samples freely settling in ultrapure water for 7 days. From left to right are blank, PCN, and K-PCN_-5.0_.


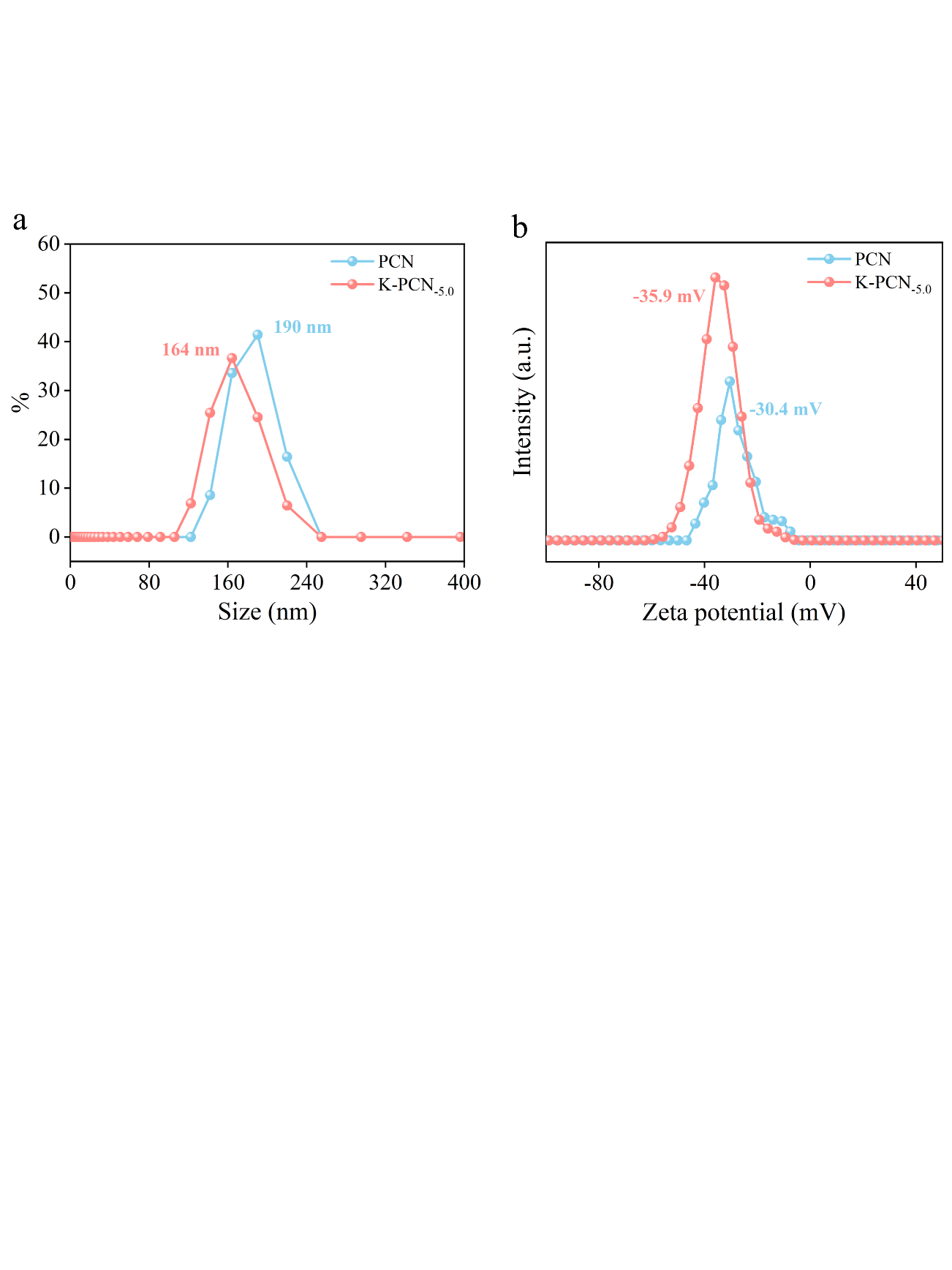


# Figure S5. (a) Hydrodynamic size distribution and (b) zeta potential of K-PCN_-5.0_ and PCN.


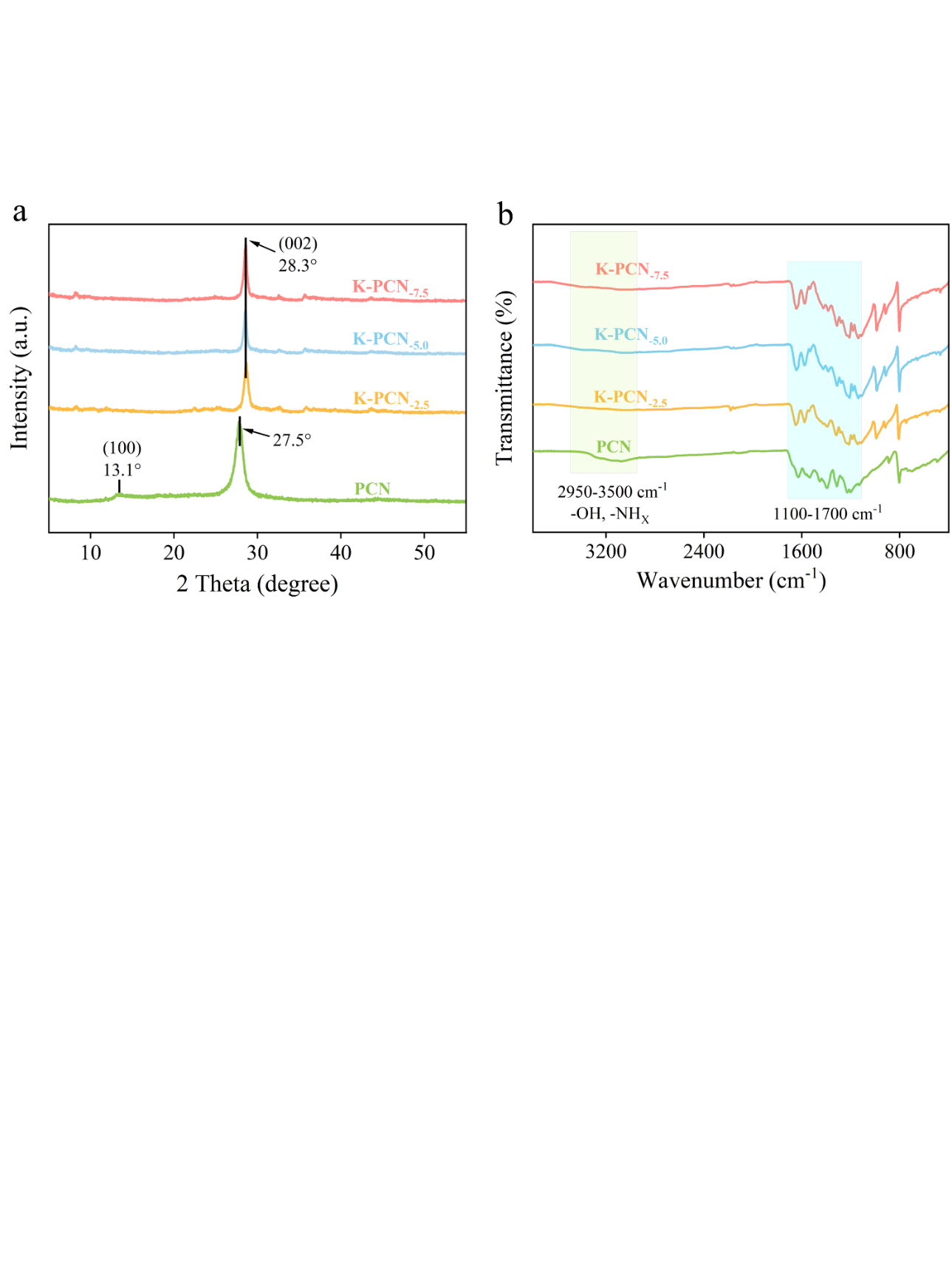


# Figure S6. (a) XRD and (b) FTIR of prepared samples.


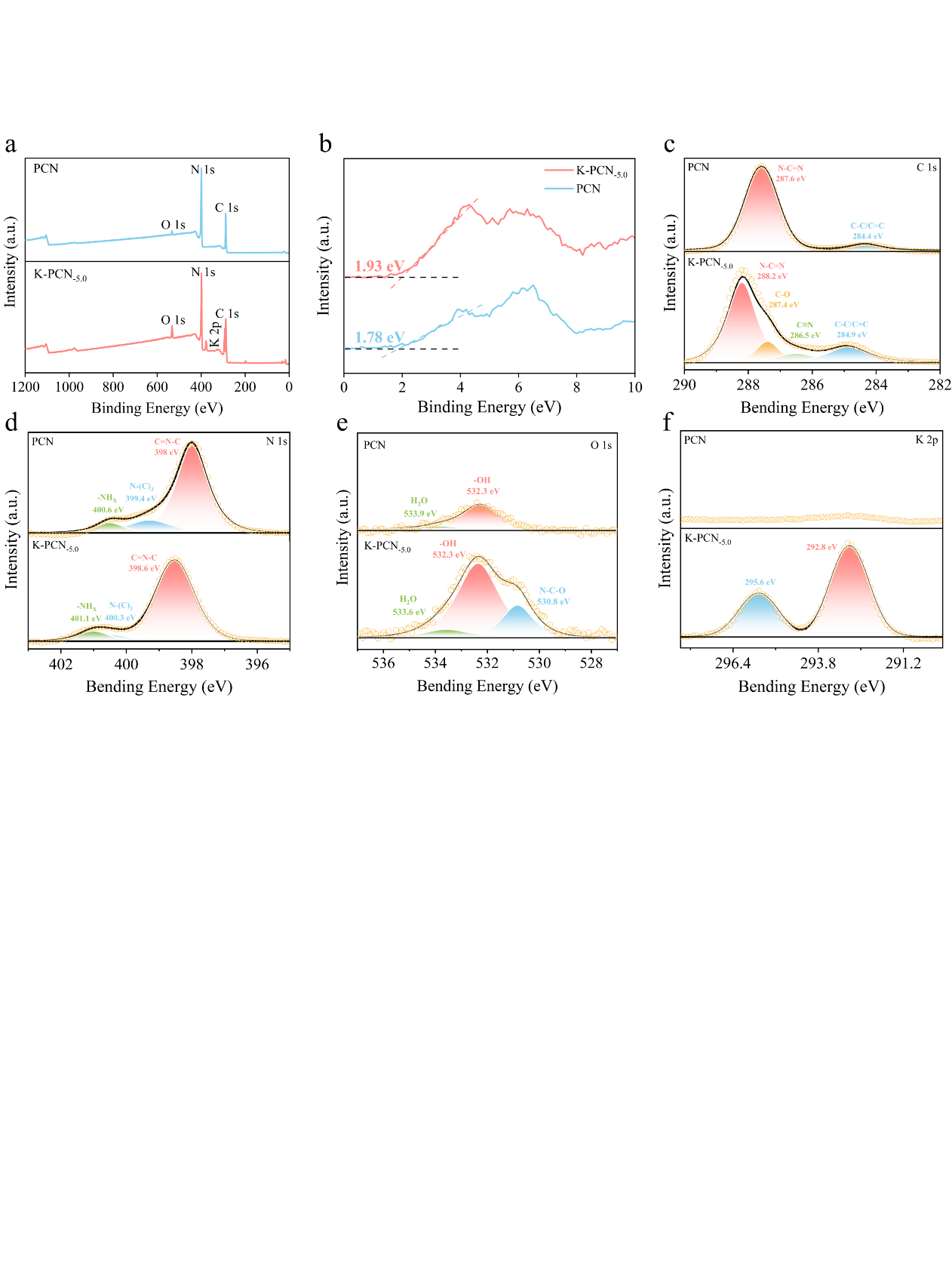


# Figure S7. (a) XPS survey and (b) XPS spectra valence band (VB) positions of PCN and K-PCN_-5.0_; High-resolution XPS spectra of (c) C 1*s*, (d) N 1*s*, (e) O 1*s*, and (i) K 2*p* of PCN and K-PCN_-5.0_.


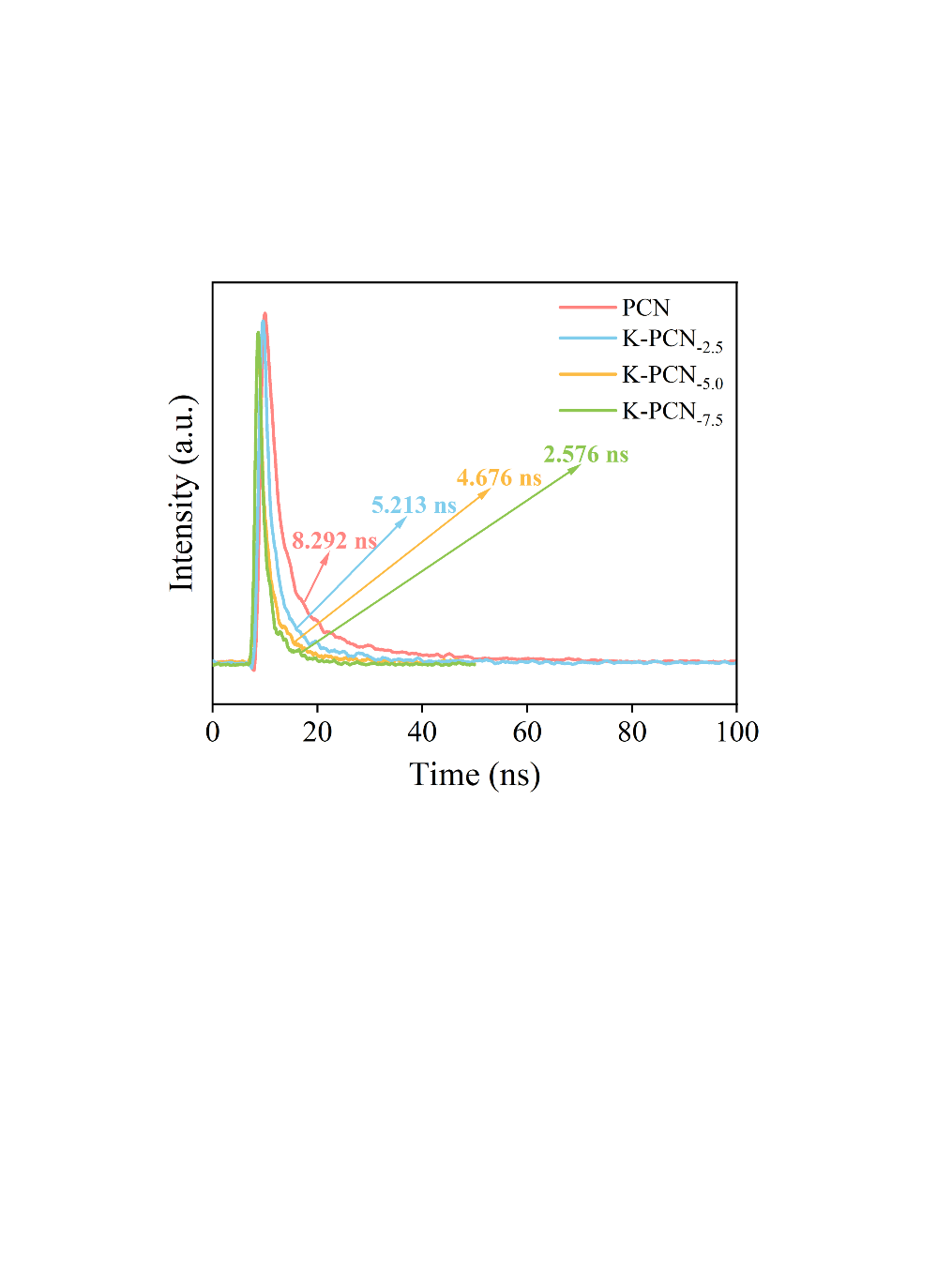


# Figure S8. Time-resolved PL spectra of prepared samples.


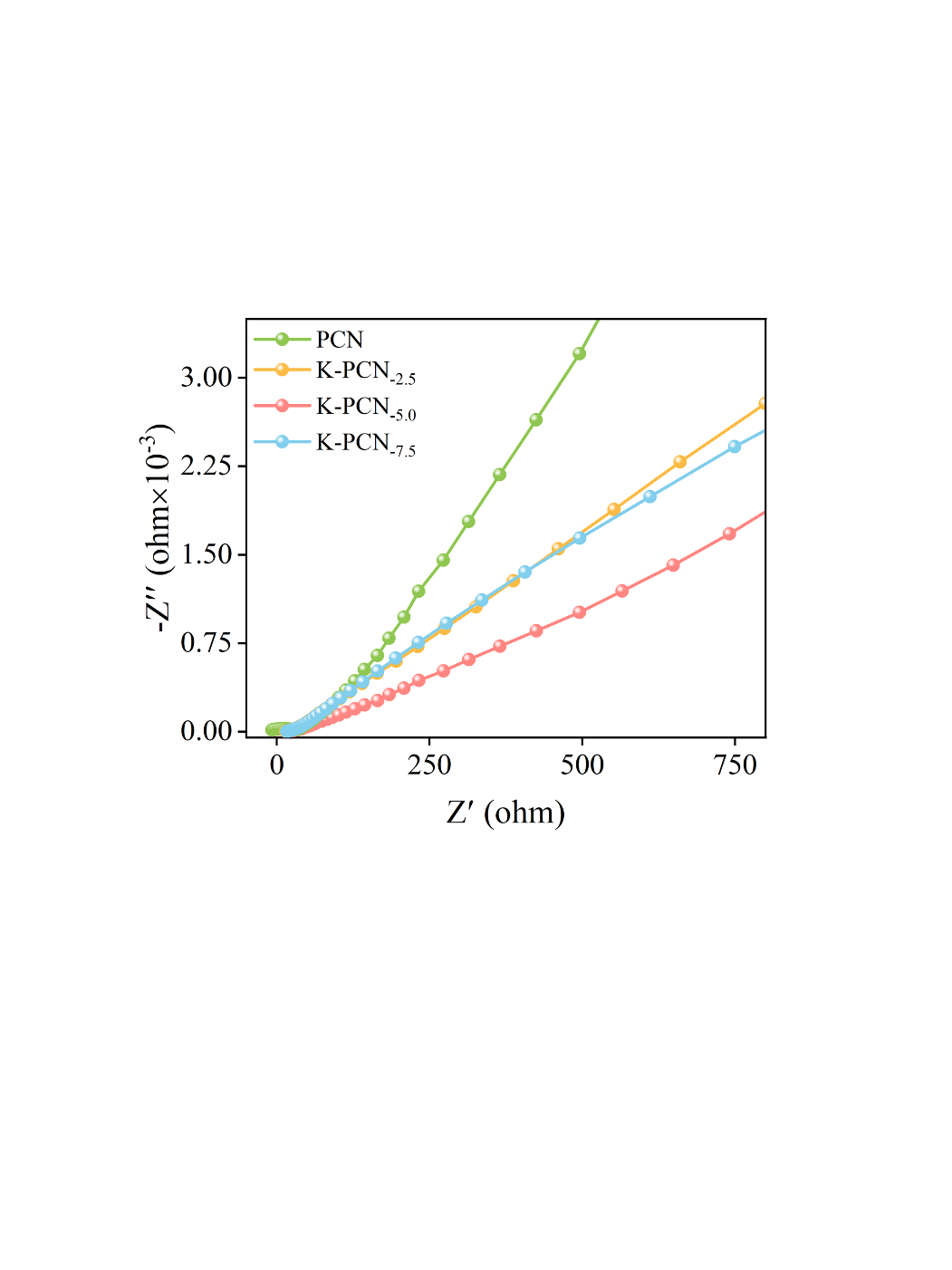


# Figure S9. EIS and transient photocurrent responses of PCN and K-PCN.


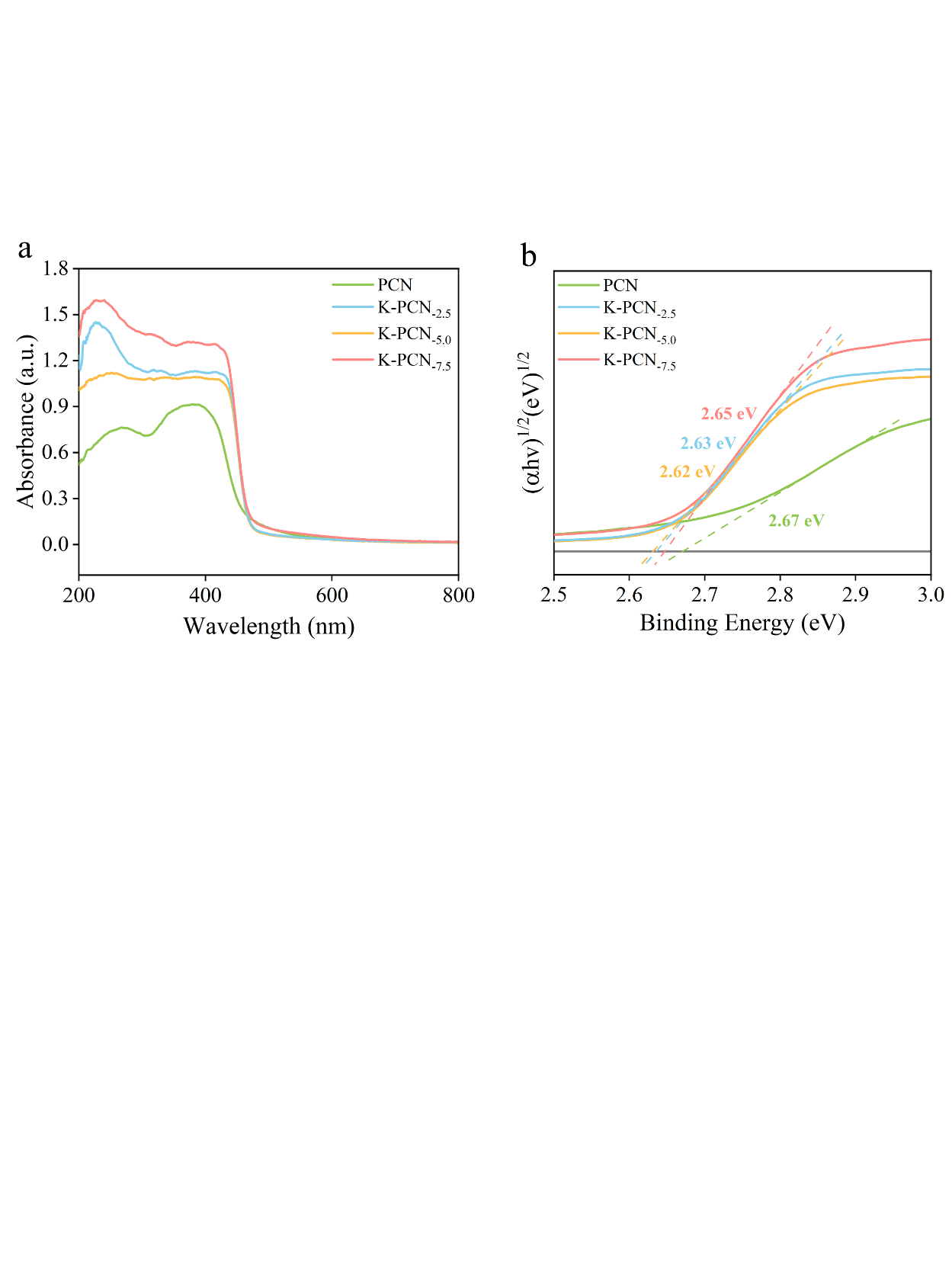


# Figure S10. (a) UV-Vis DRS and (b) band-gap analysis diagram of PCN and K-PCN.


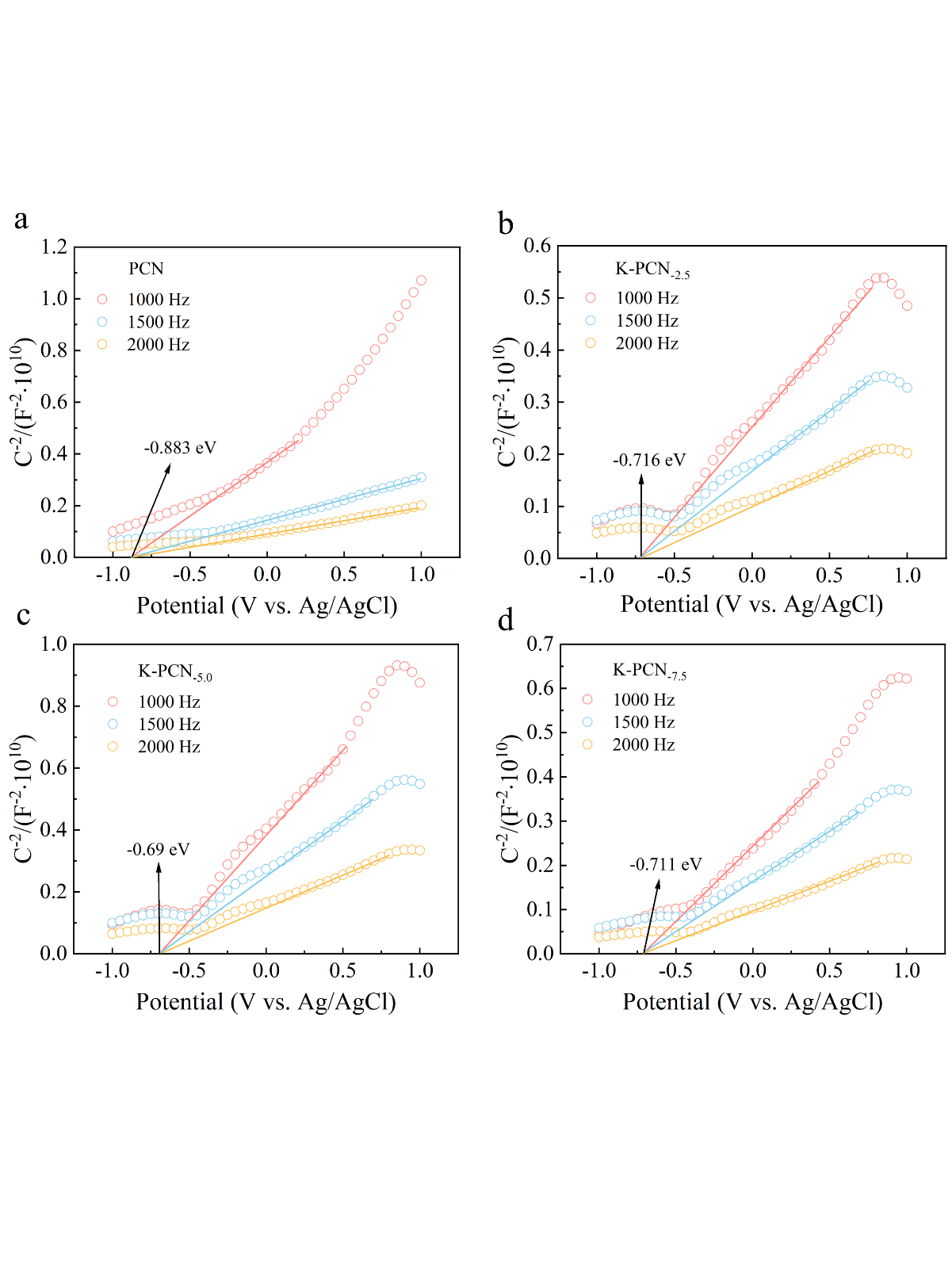


# Figure S11. Mott-Schottky curves of (a) PCN, (b) K-PCN_-2.5_, (c) K-PCN_-5.0_, and (d) K-PCN_-7.5_.


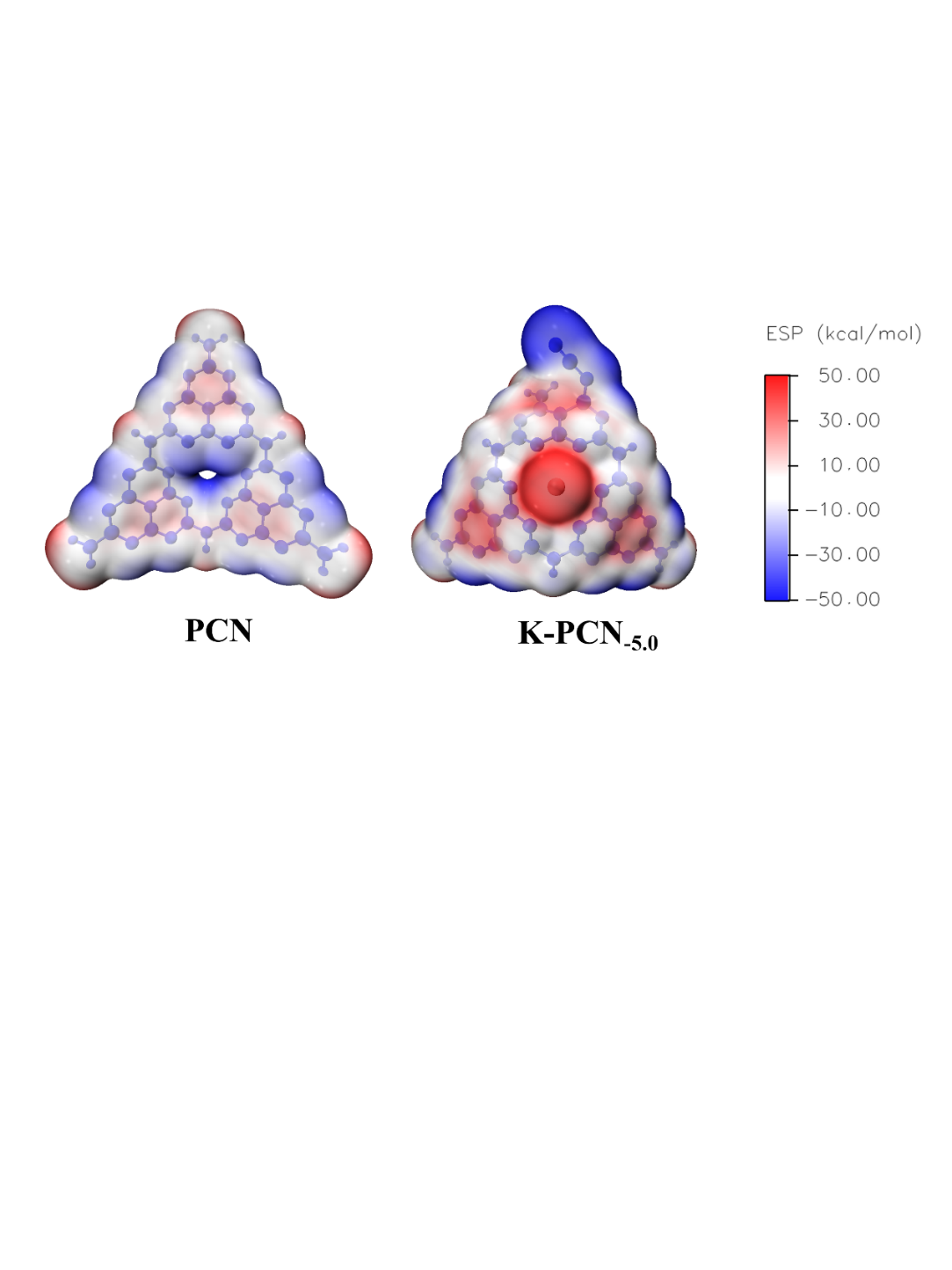


# Figure S12. Electrostatic potential surface distribution of PCN and K-PCN_-5.0_.


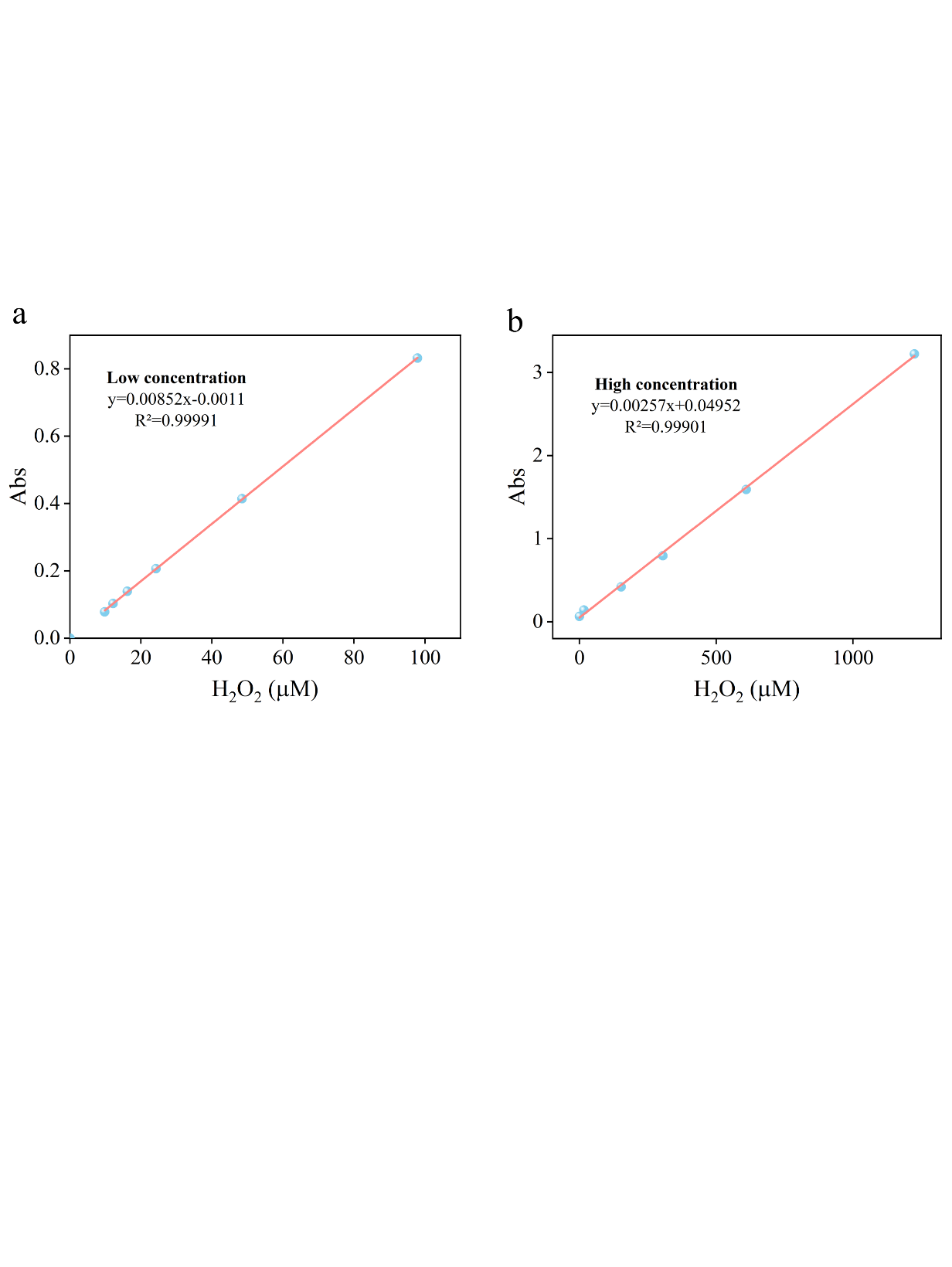


# Figure S13. Standard curves of (a) low and (b) high concentrations of H_2_O_2_ calibrated by the iodination method.


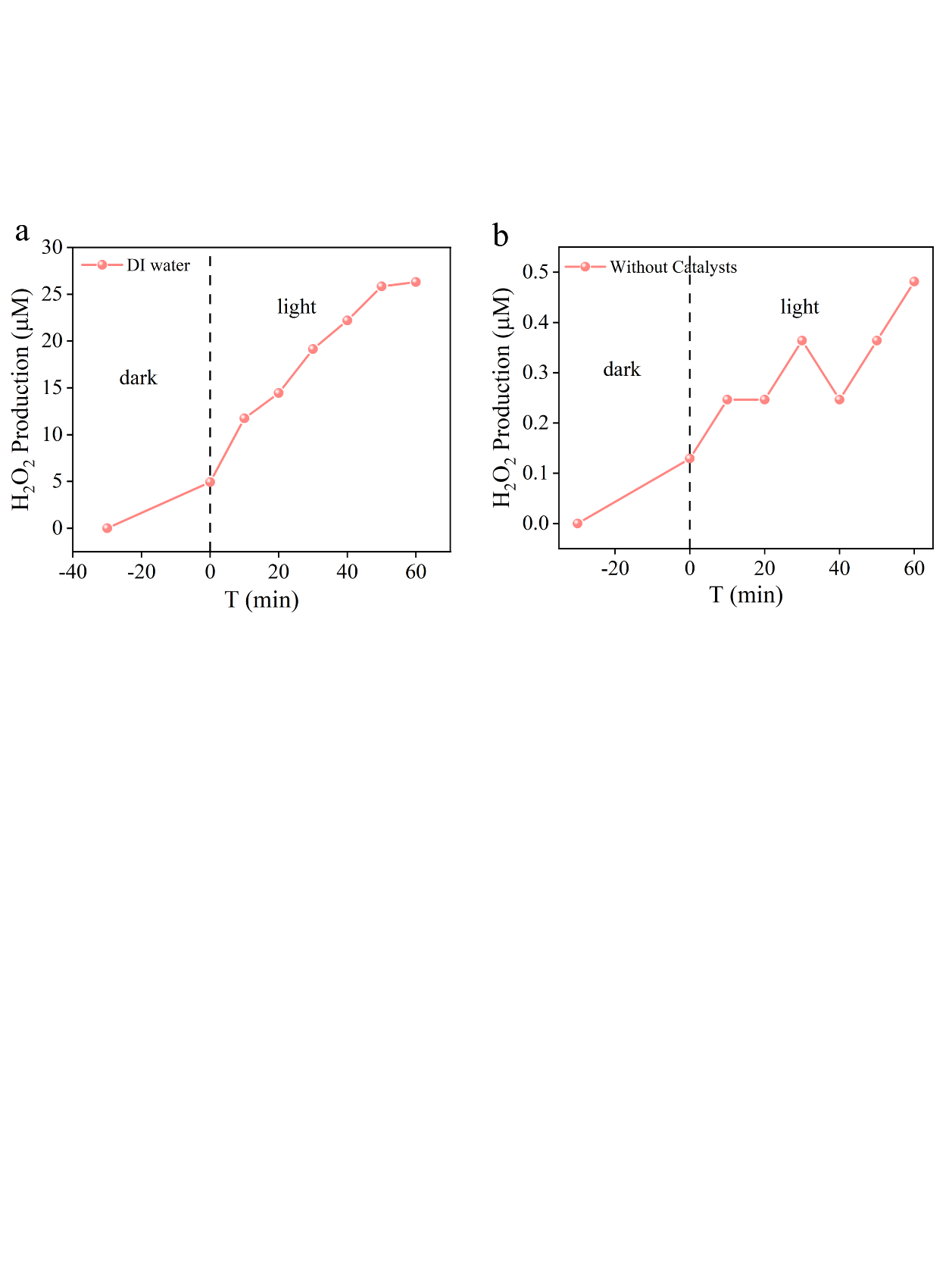


# Figure S14. (a) Photocatalytic experiments with K-PCN in the absence of a sacrificial agent; (b) Photocatalytic blank experiment without catalyst.


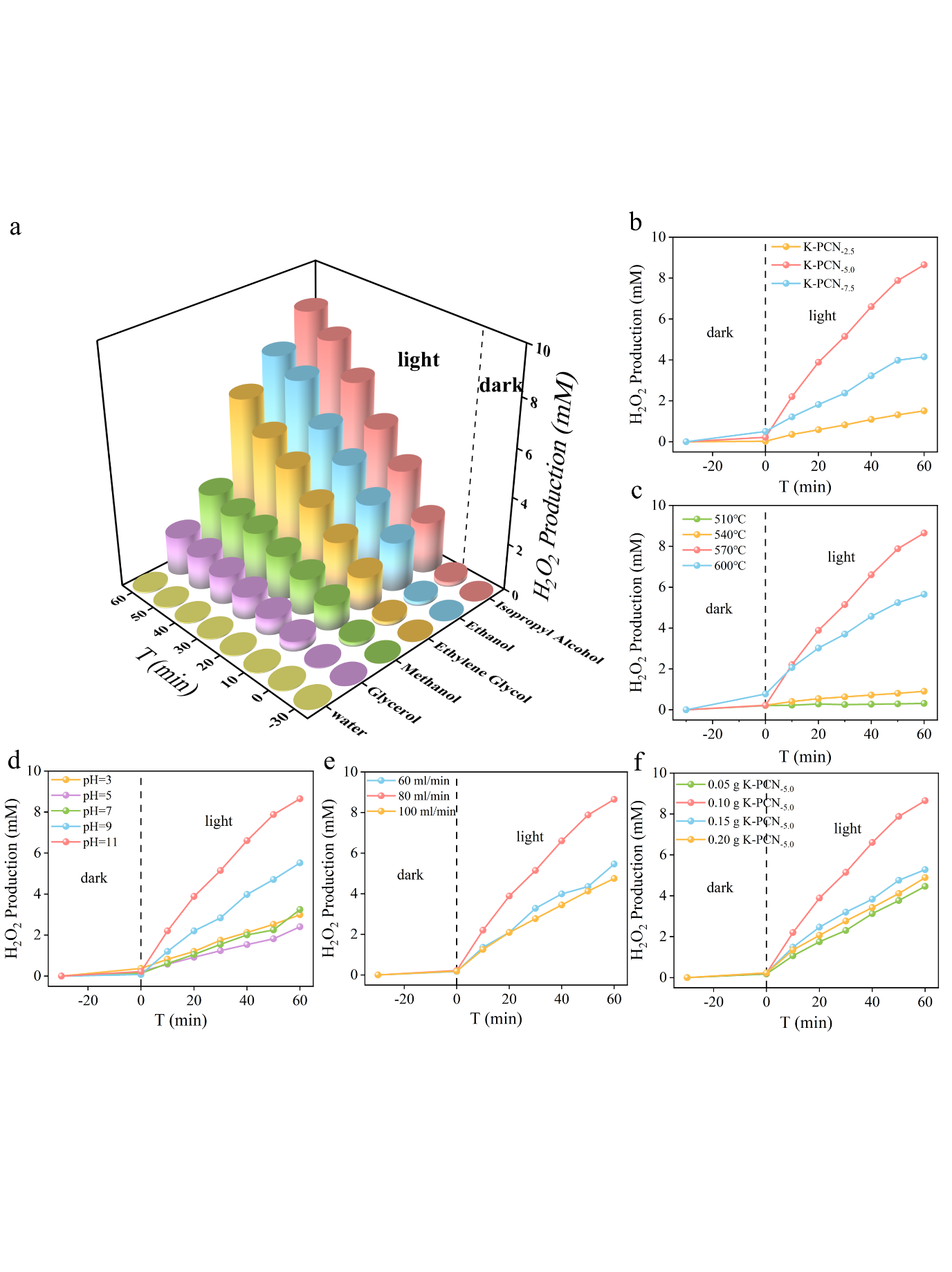


# Figure S15. Photocatalytic H_2_O_2_ production performance of K-PCN under different (a) sacrificial agents, (b) alkali metal doping ratios, (c) preparation temperatures, (d) solution pH values, (e) O_2_ fluxes, and (f) catalyst qualities.


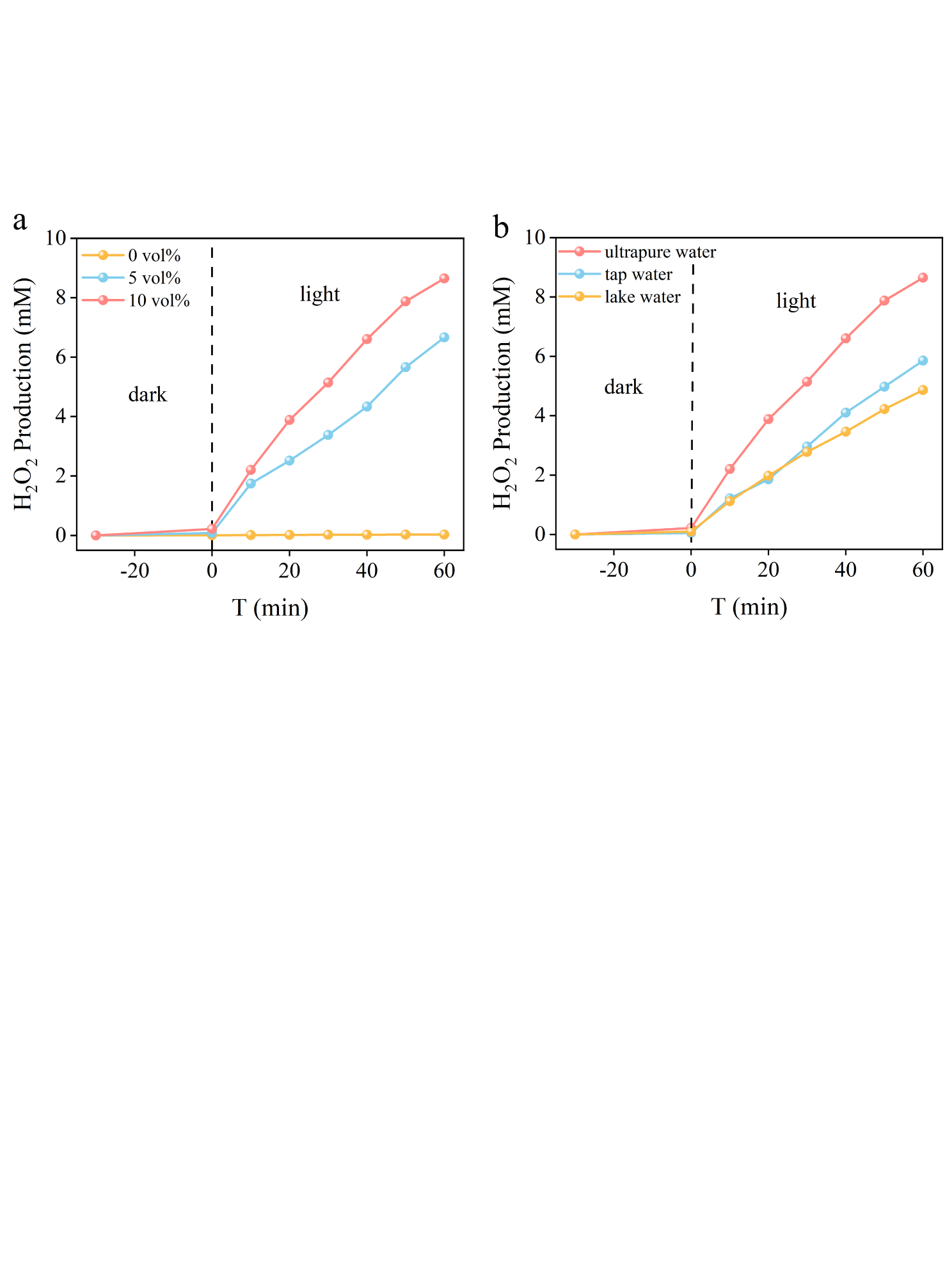


**Figure S16.** Photocatalytic H_2_O_2_ generation performance of K-PCN under (a) varying isopropyl alcohol contents and (b) different water conditions.


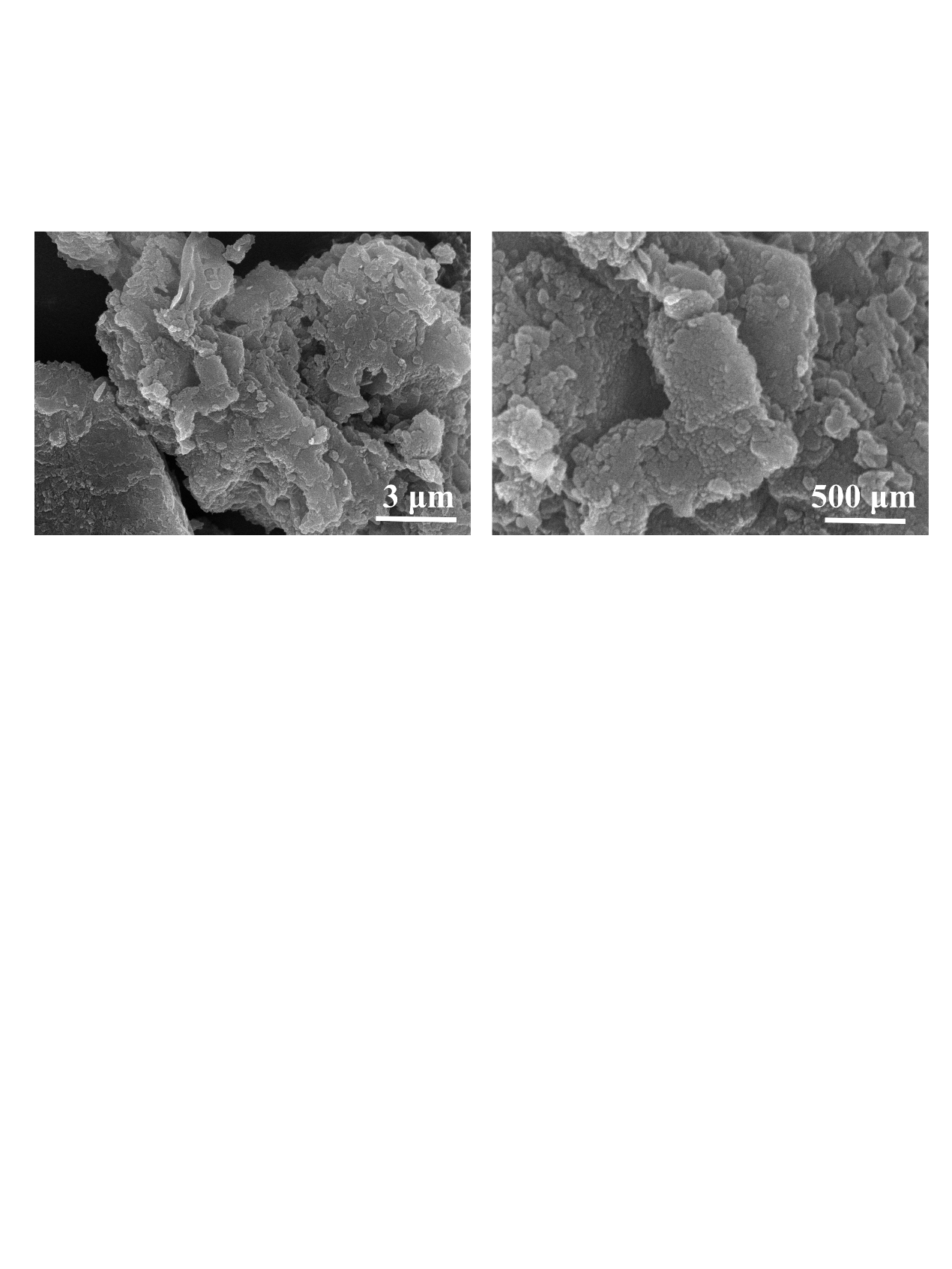


# Figure S17. SEM image of K-PCN_-5.0_ after photocatalytic reaction.


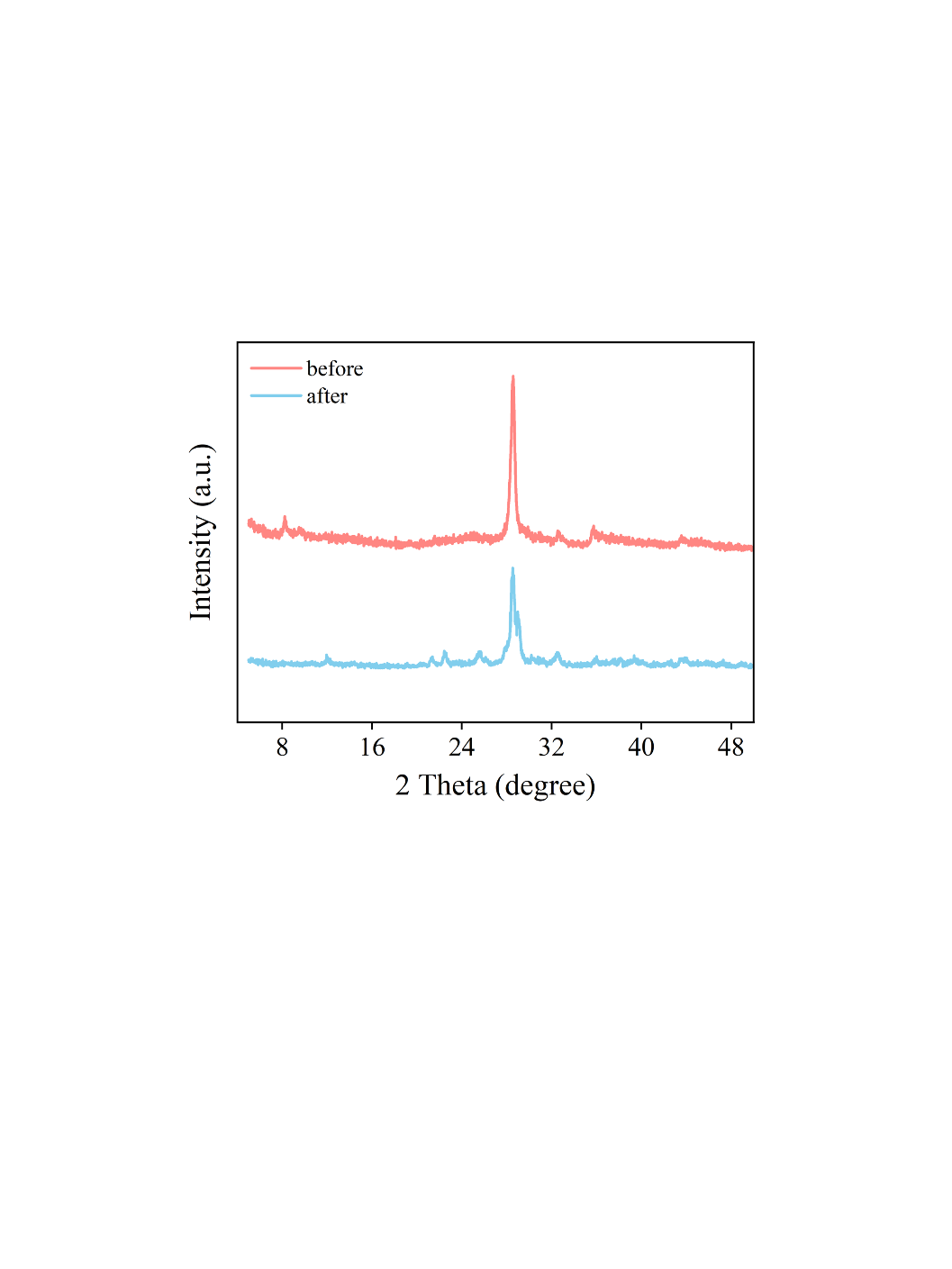


# Figure S18. XRD comparison of samples before and after photocatalytic reaction.


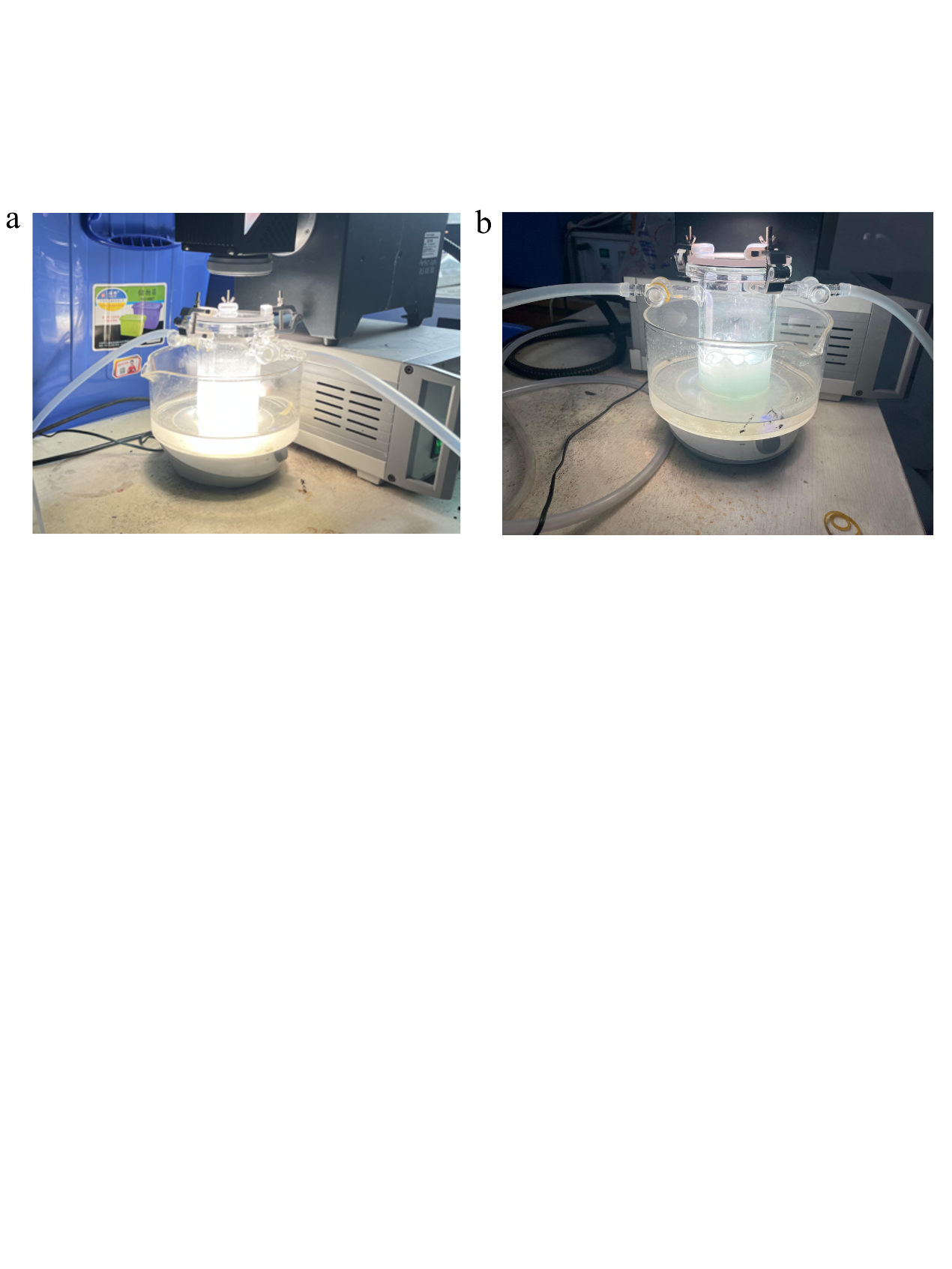


# Figure S19. Photocatalytic experiments when (a) O_2_ and (b) N_2_ were introduced.


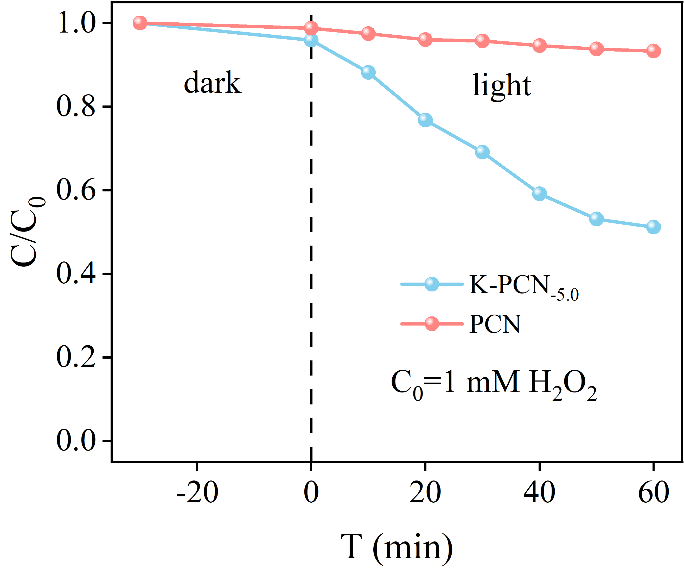


# Figure S20. H_2_O_2_ decomposition properties of different samples in N_2_ atmosphere.


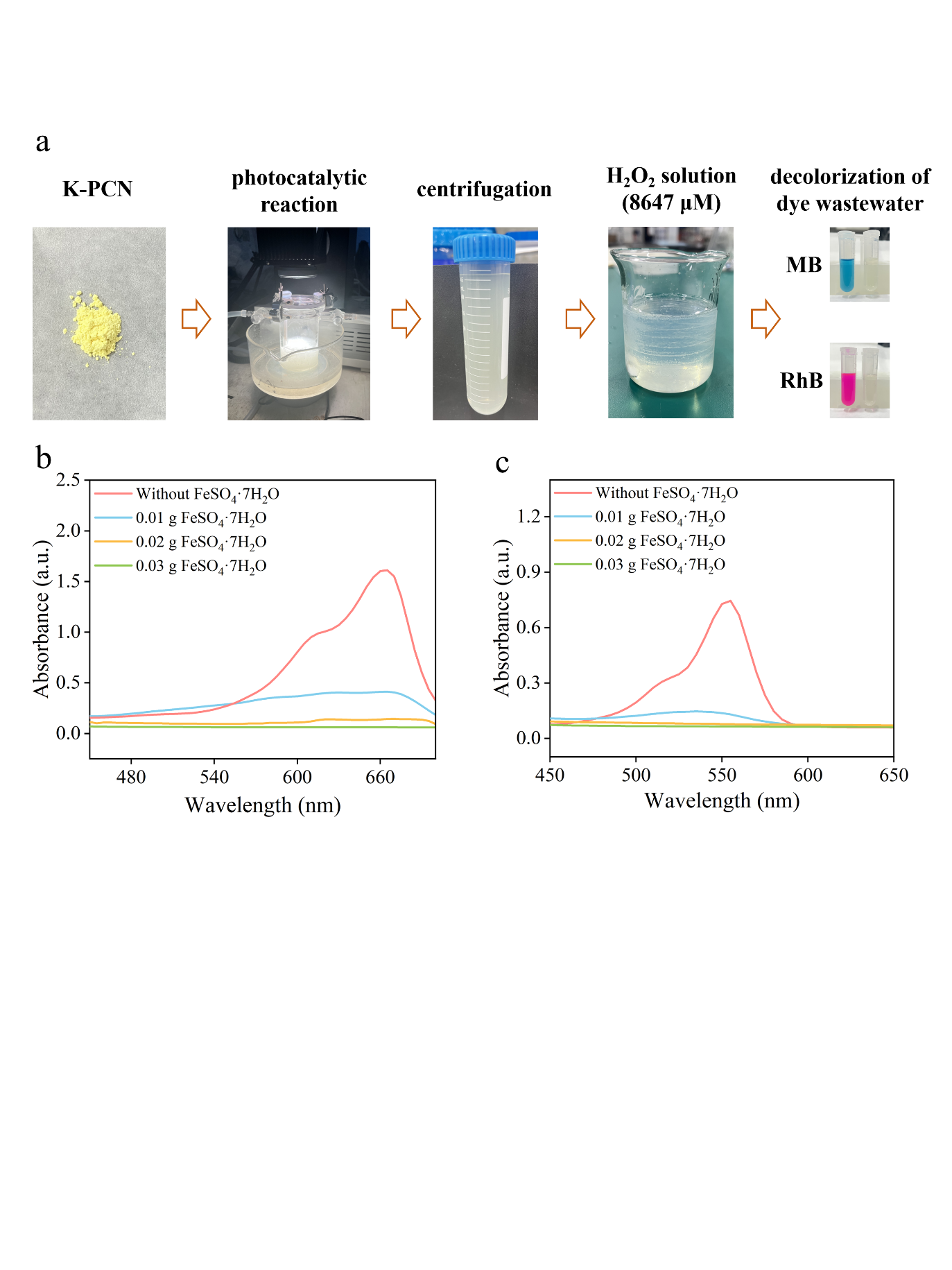


# Figure S21. (a) K-PCN_-5.0_ photocatalyst was used for photocatalytic reaction to generate H_2_O_2_ and the generated H_2_O_2_ was used for decolorization of dye wastewater. Full wavelength scanning of (b) MB and (c) RhB solutions.


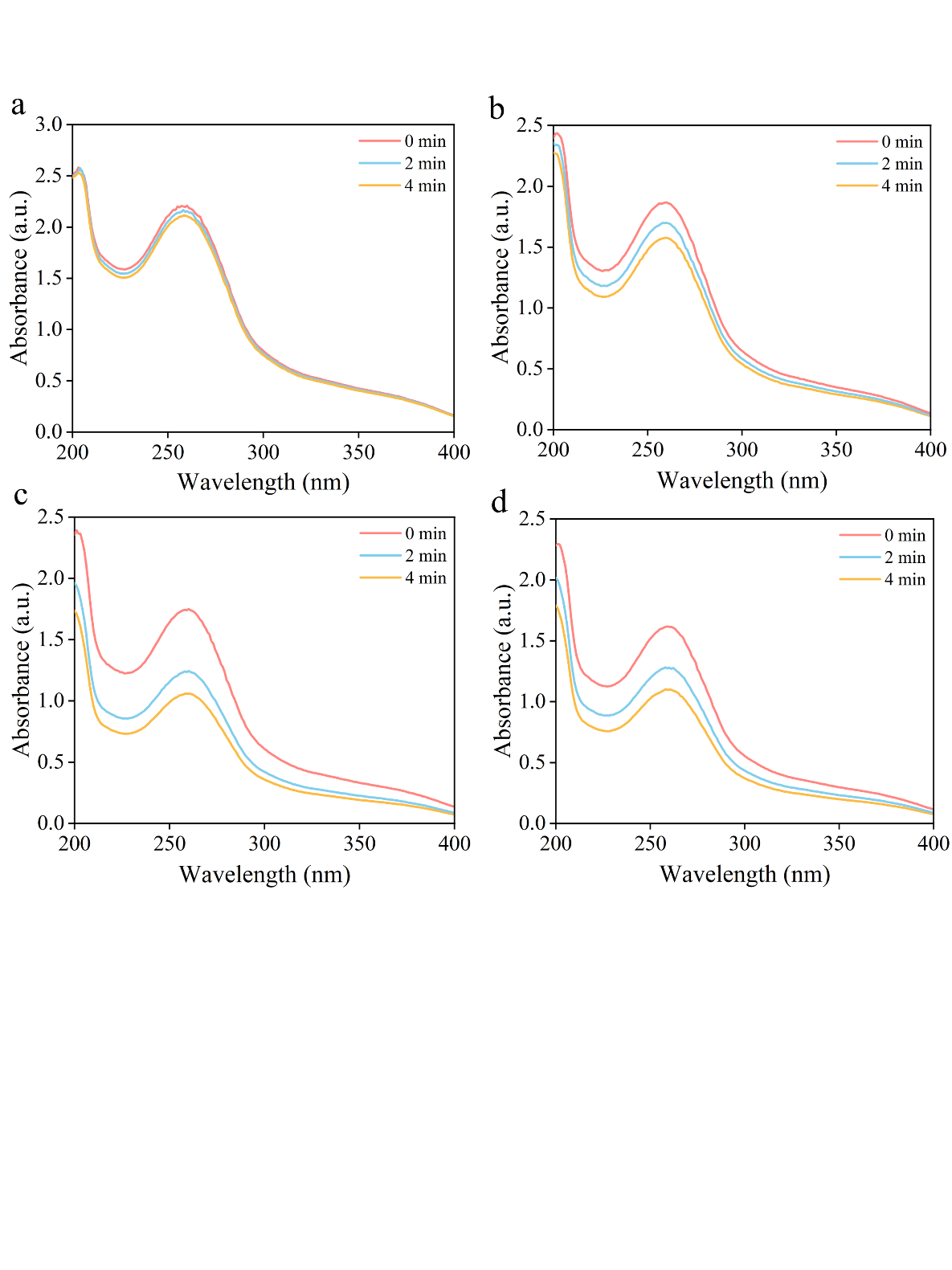


# Figure S22. The absorption intensity of NBT at 259 nm in (a) PCN, (b) K-PCN_-2.5_, (c) K-PCN_-5.0_, and (d) K-PCN_-7.5_ reaction solutions.


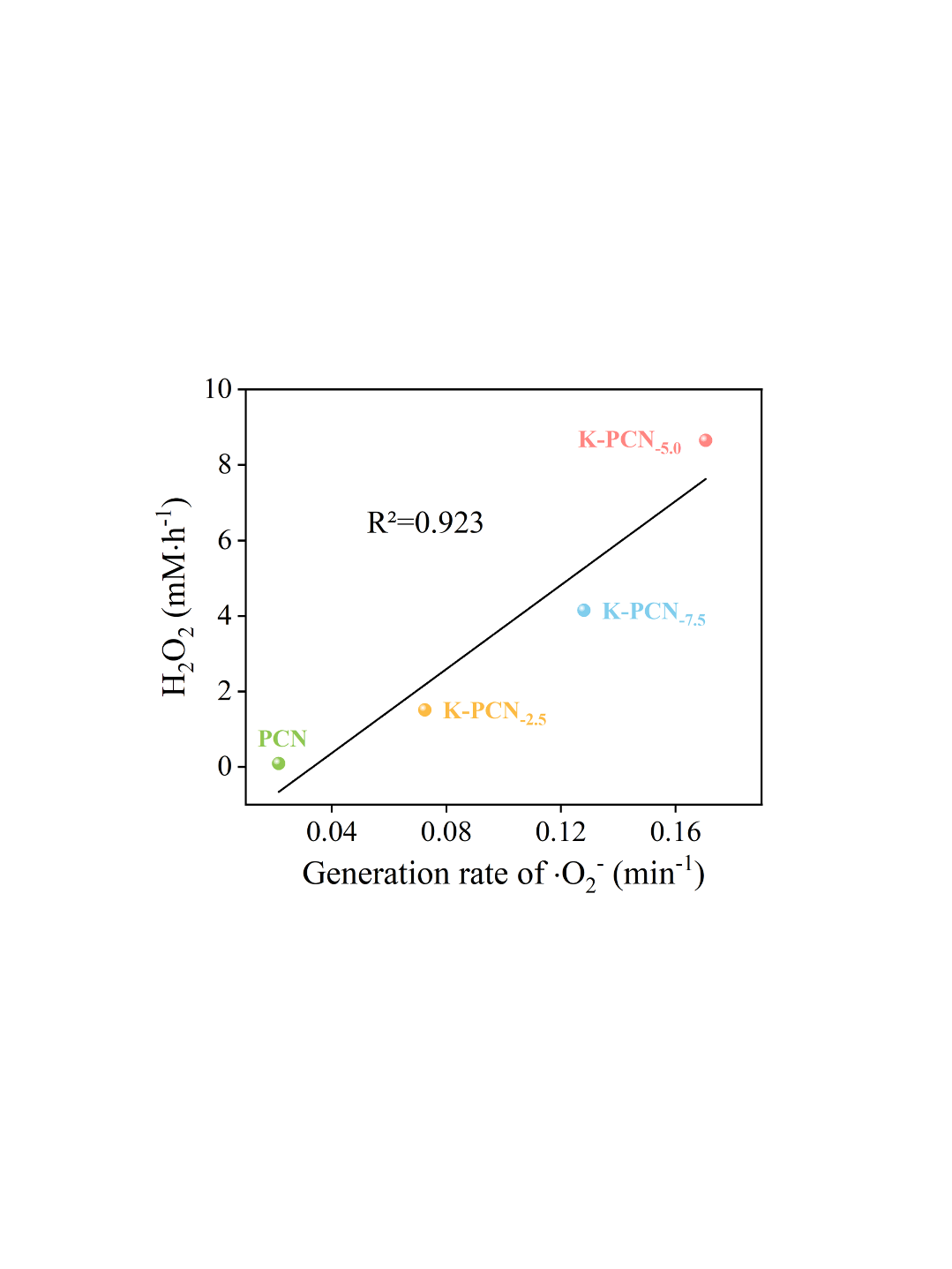


# Figure S23. Relationship between H_2_O_2_ generation rate and the •O_2_^-^ intermediate.


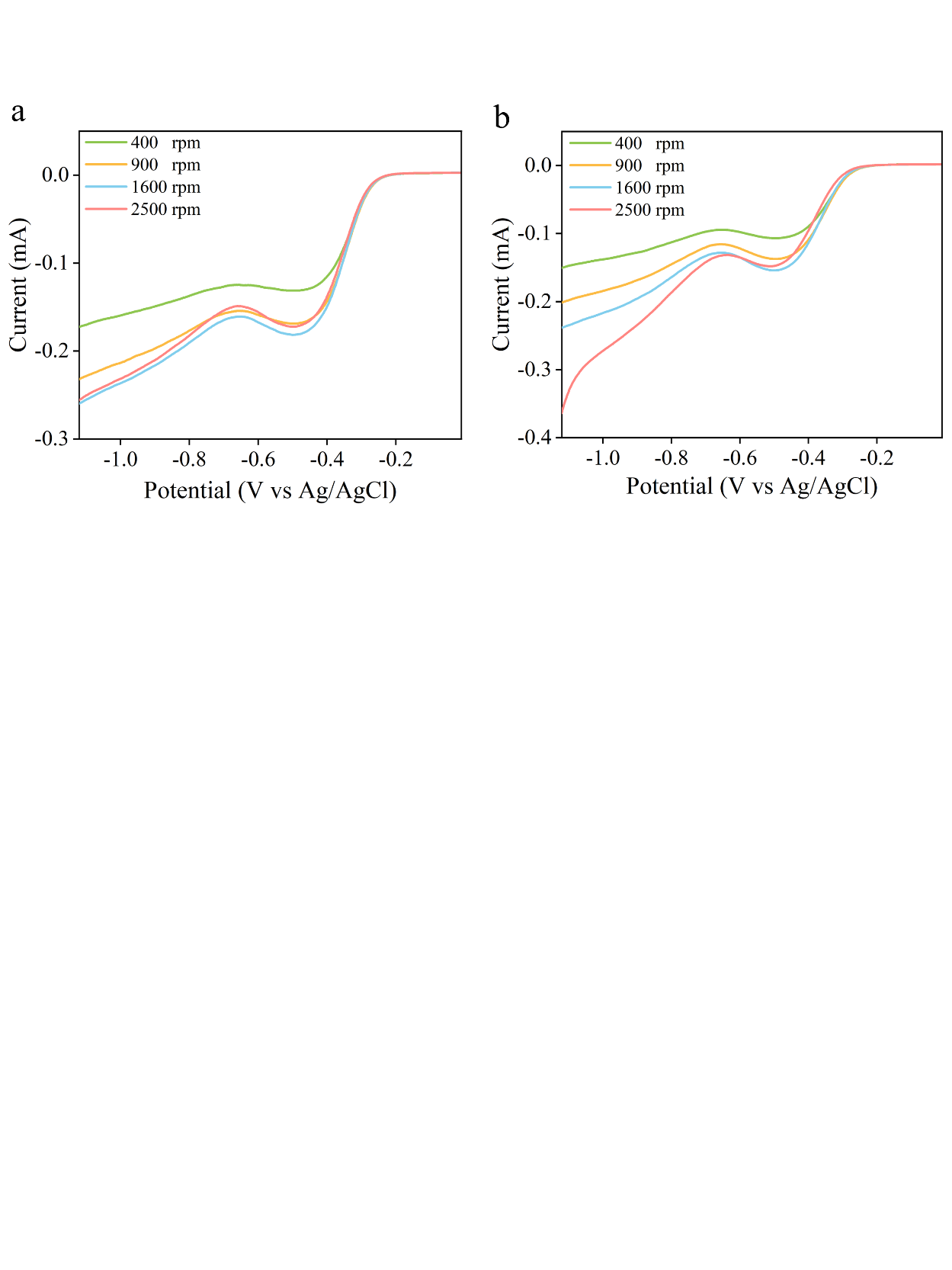


# Figure S24. RRDE polarization curves of (a) PCN and (b) K-PCN_-5.0_ in O_2_-saturated KOH (0.1 M) solution at different rotational speeds.


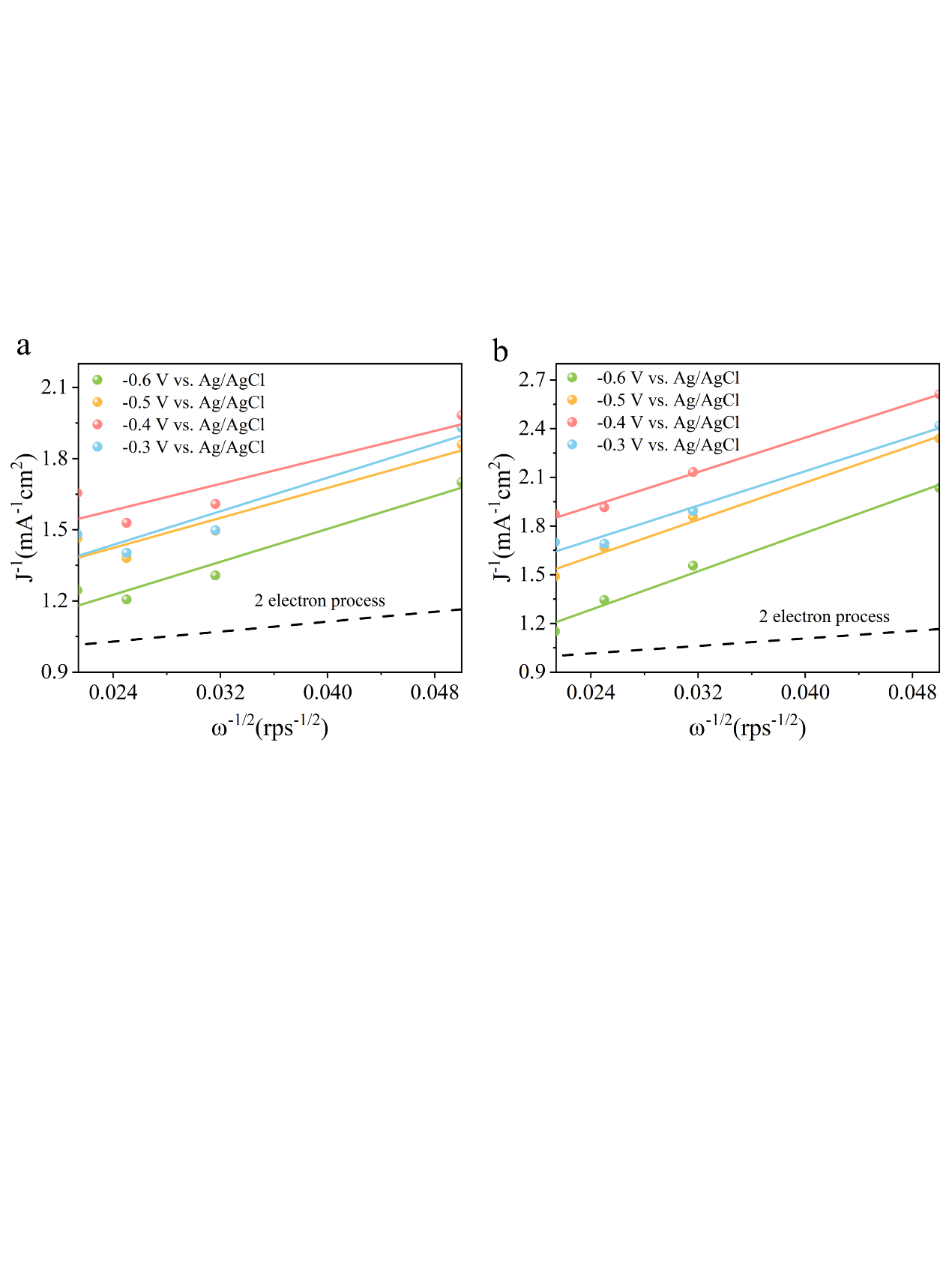


# Figure S25. Electron transfer numbers of (a) K-PCN_-5.0_ and (b) PCN fitted by the K-L equation.


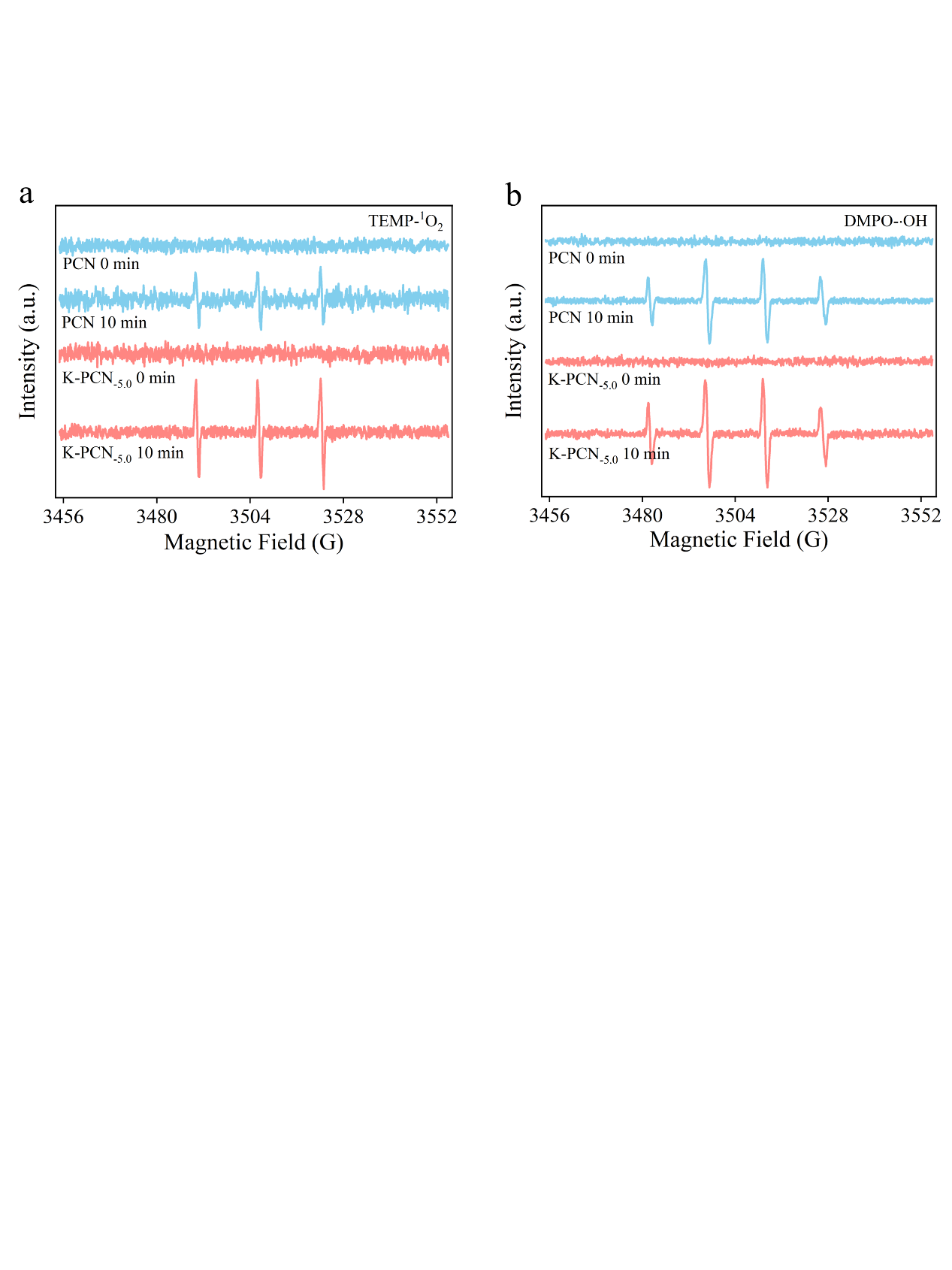


# Figure S26.The signal of (a) ^1^O_2_-TEMP and (b) •OH-DMPO.


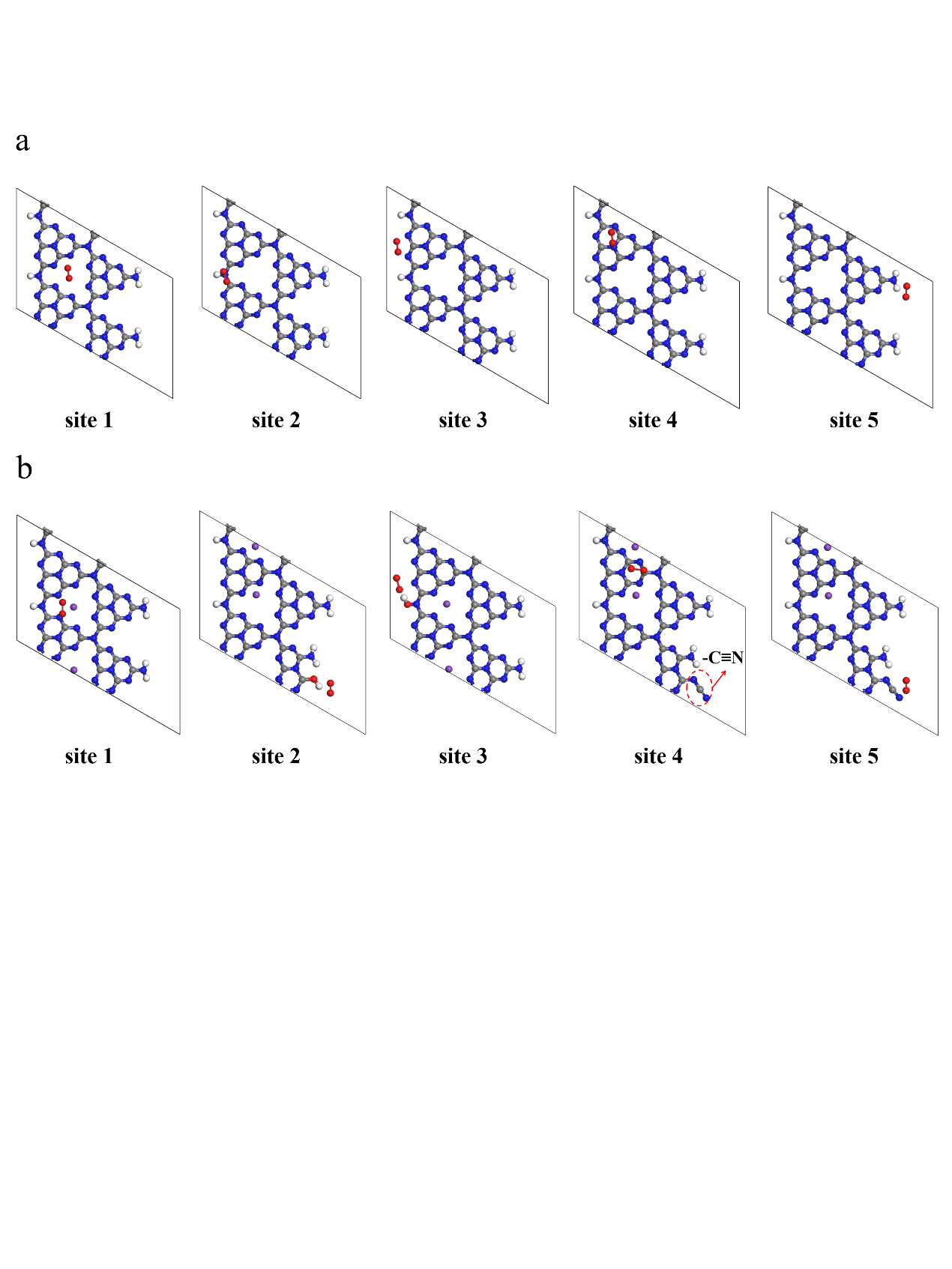


# Figure S27. Calculation of oxygen adsorption sites of simulated (a) PCN and (b) K-PCN_-5.0_.


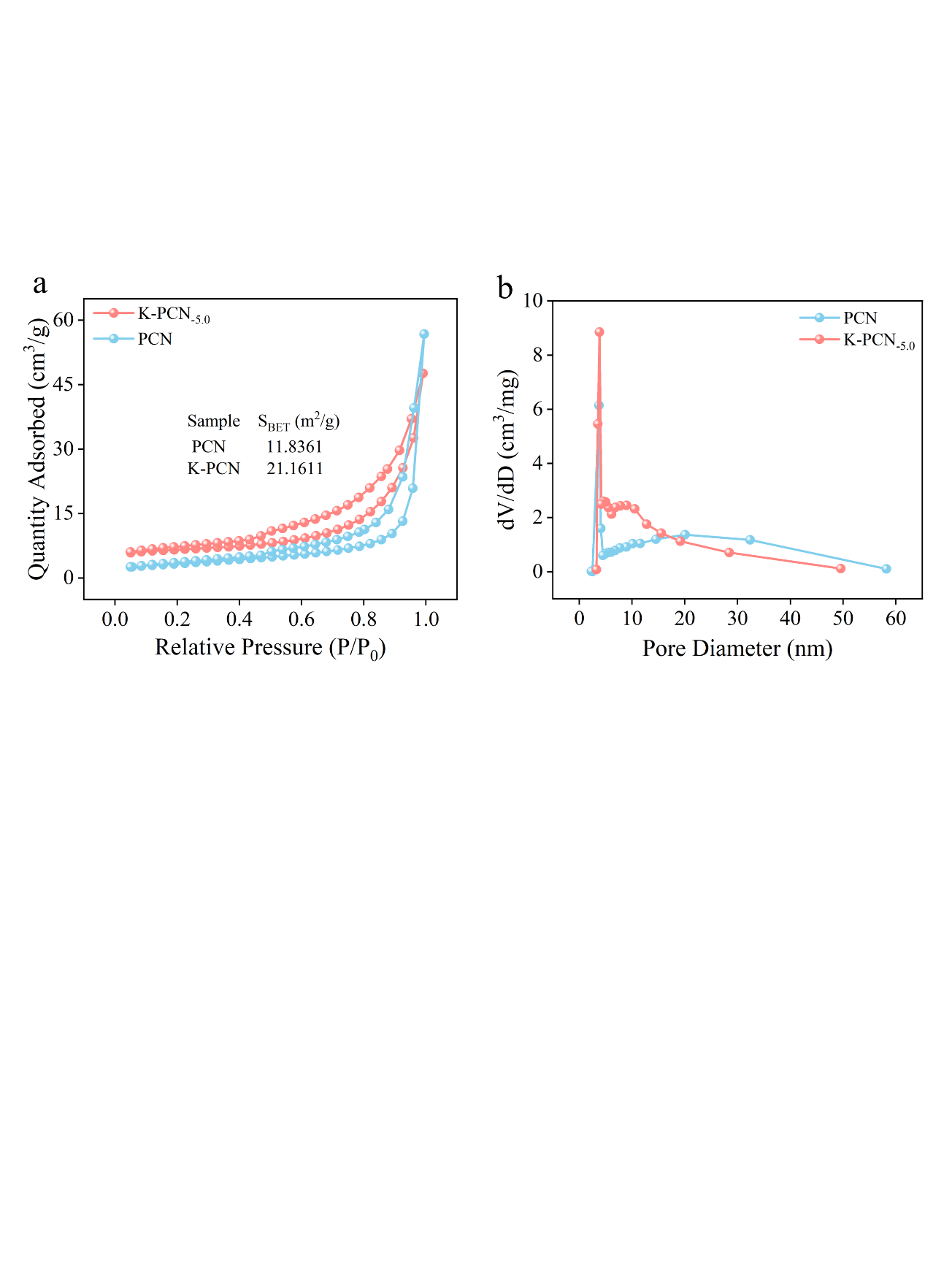


# Figure S28. (a) The nitrogen adsorption-desorption isotherm and specific surface area (insert) of prepared samples. (b) Pore size distribution of prepared samples.

# Table. S1. The values of TRPL lifetime τ_i_ (E_λ_ = 360 nm) and corresponding constant A_i_.

| Samples | A_1_ (%) | τ_1_ (ns) | A_2_ (%) | τ_2_ (ns) | τ_ave_ (ns) |
| --- | --- | --- | --- | --- | --- |
| PCN | 0.78806 | 2.61554 | 0.16299 | 13.57716 | 8.292 |
| K-PCN_-2.5_ | 0.92316 | 1.7672 | 0.12756 | 9.73287 | 5.213 |
| K-PCN_-5.0_ | 0.77764 | 1.33613 | 0.20462 | 7.07128 | 4.676 |
| K-PCN_-7.5_ | 0.67922 | 0.92672 | 0.18749 | 3.96902 | 2.576 |

The values of TRPL lifetime was calculated using the following equation:

 （1）

 （2）

where, A_1_ and A_2_ are the preexponential factors, τ_1_ and τ_2_ represent the long and short fluorescent lifetimes.

# Table. S2. Conversion of reference electrode potentials for samples.

| Samples  Reference electrode | PCN | K-PCN_-2.5_ | K-PCN_-5.0_ | K-PCN_-7.5_ |
| --- | --- | --- | --- | --- |
| .Ag/AgCl | -0.883 | -0.716 | -0.69 | -0.711 |
| .NHE | -0.886 | -0.719 | -0.693 | -0.714 |

The band edge positions of samples can be calculated suing the following equation:

 （3）

 （4）

E_VB_ and E_CB_ stand for the valence band edge potential and conduction band edge potential, respectively; X is the voltage different between the conduction band value and the flat potential value, generally 0.1-0.2 eV, which is set as 0.2 eV in this work.

# Table. S3. Parameters for calculating AQY for H_2_O_2_ production by K-PCN_-5.0_ photocatalysis.

| Wavelength (nm) | Light intensity (mW·cm^-1^) | H_2_O_2_ yields (mM) | AQY (%) |
| --- | --- | --- | --- |
| 420 | 23.2 | 7.42 | 17.92 |
| 450 | 23.8 | 6.82 | 14.97 |
| 475 | 24.8 | 1.73 | 3.45 |
| 500 | 22.5 | 0.59 | 1. 23 |
| 520 | 22.6 | 0.4 | 0.8 |
| 550 | 23.1 | 0.47 | 0.87 |
| 580 | 28.9 | 0.34 | 0.48 |

# Table. S4. Parameters for calculating AQY for H_2_O_2_ production by PCN photocatalysis.

| Wavelength (nm) | Light intensity (mW·cm^-1^) | H_2_O_2_ yields (mM) | AQY (%) |
| --- | --- | --- | --- |
| 420 | 23.2 | 0.0533 | 0.129 |
| 450 | 23.8 | 0.0344 | 0.076 |
| 475 | 24.8 | 0.0111 | 0.022 |
| 500 | 22.5 | 0.0058 | 0.012 |
| 520 | 22.6 | 0.0091 | 0.018 |
| 550 | 23.1 | 0.0091 | 0.017 |
| 580 | 28.9 | 0.0056 | 0.0079 |

The apparent quantum efficiency (AQY) of the sample was calculated using the following equation:

 （5）

M represents the amount of produced H_2_O_2_ molecules (mol), N_A_ is the Avogadro constant (6.02×10^23^ mol^-1^), h represents the Planck constant (6.63×10^-34^ J s), c is the speed of light (3×10^8^ m s^-1^), S is the irradiation area (cm^2^), P is the intensity of irradiation light (mW cm^-2^), t is the photoreaction time (s), and λ is the wavelength of the incident monochromatic light (nm).

# Table. S5. Comparison of the performance of photocatalytic H_2_O_2_ production in the reported literature.

| Samples | Catalyst dose | Reaction solution | Light source | H_2_O_2_ rate (mM·h^-1^) | Refs. |
| --- | --- | --- | --- | --- | --- |
| Zn_0.5_Cd_0.5_S | 0.3 g/L | No scavenger | 300 W Xe lamp (λ≥420 nm) | 0.041 | [1] |
| CN-DCCNa | 1.0 g/L | ethanol (10%) | 300 W Xe lamp (λ≥420 nm) | 0.258 | [2] |
| g-C_3_N_4_  nanosheet | 0.5 g/L | isopropanol (10%) | 300 W Xe lamp (λ≥420 nm) | 0.567 | [3] |
| Cyano-g-C_3_N_4_/In_2_S_3_/Ppy | 1.0 g/L | isopropanol (10%) | 300 W Xe lamp (λ≥420 nm) | 0.895 | [4] |
| FL-CN-530 | 0.5 g/L | ethanol (5%) | 300 W Xe lamp (λ≥420 nm) | 0.952 | [5] |
| AKCN | 0.5 g/L | ethanol (10 %) | 300 W Xe lamp (λ≥420 nm) | 1.133 | [6] |
| Nv-C≡N-CN | 1.0 g/L | isopropanol (10%) | 300 W Xe lamp (λ≥420 nm) | 3.093 | [7] |
| TP-PCN | 0.5 g/L | isopropanol (10%) | 300 W Xe lamp (λ≥420 nm) | 3.265 | [8] |
| NVCNS | 1.0 g/L | isopropanol (10%) | 300 W Xe lamp (λ≥420 nm) | 4.413 | [9] |
| Nv-g-C_3_N_4_ | 0.25 g/L | ethanol (25%) | AM 1.5G | 7.63 | [10] |
| IO-CN-Cv | 1.0 g/L | ethanol (5%) | 300 W Xe lamp (λ≥420 nm) | 8.143 | [11] |
| **K-PCN** | **1.0 g/L** | **isopropanol (10%)** | **300 W Xe lamp (λ≥420 nm)** | **8.65** | **This work** |

# Table. S6. The number of transferred electrons for K-PCN_-5.0_ and PCN fitted by the K-L equation.

| Samples | -0.6 V | -0.5 V | -0.4 V | -0.3 V |
| --- | --- | --- | --- | --- |
| K-PCN | 1.6 | 1.75 | 1.99 | 1.57 |
| PCN | 0.94 | 0.97 | 1.05 | 1.04 |

The numbers of electron transfer (𝑛) were obtained by the Koutecky-Levich equation:

 （6）

𝑖_𝐷_ is the measured current density, i_K_ is the control current, 𝐹 is the Faraday constant (96487 C mol^-1^), 𝐴 is the electrode area (cm^2^), 𝐷 is the diffusion coefficient of the oxygen (1.93×10^-5^ cm^2^ s^-1^), 𝜈 is the kinematic viscosity of the solution (0.0109 cm^2^ s^-1^), 𝐶 is the bulk concentration of oxygen (1.26×10^-3^ M), and 𝑛 is an overall number of transferred electrons, 𝜔 is the angular rotation rate.

**References**

[1] Y. Wang, M. Zhang, H. Wu, M. Huang, Y. Fang, M. Anpo, X. Wang, Si/Carbon-dots with Surface N-C Sites Promoting Proton and Electron Transfers in Oxygen Reduction Reaction, Angew Chem Int Ed, 64 (2025) e202509790.

[2] H. Zhang, Z. Zhu, Y. Sun, L. Han, Organic Sodium Salt-Assisted Synthesis of Multi-Defective Highly Dense Structural Carbon Nitride for Efficient Photocatalytic H_2_O_2_ Production, Small, (2025) e10119.

[3] C. Feng, L. Tang, Y. Deng, J. Wang, Y. Liu, X. Ouyang, H. Yang, J. Yu, J. Wang, A novel sulfur-assisted annealing method of g-C_3_N_4_ nanosheet compensates for the loss of light absorption with further promoted charge transfer for photocatalytic production of H_2_ and H_2_O_2_, Appl Catal, B, 281 (2021).

[4] R. Li, K. Ba, D. Zhang, Y. Shi, C. Li, Y. Yu, M. Yang, Unraveling the Synergistic Mechanism of Boosted Photocatalytic H_2_O_2_ Production over Cyano-g-C_3_N_4_/In_2_S_3_/Ppy Heterostructure and Enhanced Photocatalysis-Self-Fenton Degradation Performance, Small, 29 (2024) 2308568.

[5] B. Feng, Y. Liu, K. Wan, S. Zu, Y. Pei, X. Zhang, M. Qiao, H. Li, B. Zong, Tailored Exfoliation of Polymeric Carbon Nitride for Photocatalytic H_2_O_2_ Production and CH_4_ Valorization Mediated by O_2_ Activation, Angew Chem Int Ed, 24 (2024) e202401884.

[6] P. Zhang, D. Sun, A. Cho, S. Weon, S. Lee, J. Lee, J.W. Han, D.P. Kim, W. Choi, Modified carbon nitride nanozyme as bifunctional glucose oxidase-peroxidase for metal-free bioinspired cascade photocatalysis, Nat Commun, 10 (2019) 940.

[7] X. Zhang, P. Ma, C. Wang, L. Gan, X. Chen, P. Zhang, Y. Wang, H. Li, L. Wang, X. Zhou, K. Zheng, Unraveling the dual defect sites in graphite carbon nitride for ultra-high photocatalytic H_2_O_2_ evolution, Energy Environ Sci, 15 (2022) 830-842.

[8] H. Che, X. Gao, J. Chen, J. Hou, Y. Ao, P. Wang, Iodide-Induced Fragmentation of Polymerized Hydrophilic Carbon Nitride for High-Performance Quasi-Homogeneous Photocatalytic H_2_O_2_ Production, Angew Chem Int Ed, 60 (2021) 25546-25550.

[9] Y. Zheng, Y. Luo, Q. Ruan, J. Yu, X. Guo, W. Zhang, H. Xie, Z. Zhang, J. Zhao, Y. Huang, Plasma-Tuned nitrogen vacancy graphitic carbon nitride sphere for efficient photocatalytic H_2_O_2_ production, J Colloid Interface Sci, 609 (2022) 75-85.

[10] Y. Xie, Y. Li, Z. Huang, J. Zhang, X. Jia, X.-S. Wang, J. Ye, Two types of cooperative nitrogen vacancies in polymeric carbon nitride for efficient solar-driven H_2_O_2_ evolution, Appl Catal, B, 265 (2020).

[11] M. Wang, P. Ju, J. Li, Y. Zhao, X. Han, Z. Hao, Facile Synthesis of MoS_2_/g-C_3_N_4_/GO Ternary Heterojunction with Enhanced Photocatalytic Activity for Water Splitting, ACS Sustainable Chem Eng, 5 (2017) 7878-7886.
